# Torsion Effects Beyond the δ Bond and the Role of π Metal‐Ligand Interactions

**DOI:** 10.1002/advs.202401293

**Published:** 2024-04-03

**Authors:** Almudena Inchausti, Rosa Mollfulleda, Marcel Swart, Josefina Perles, Santiago Herrero, Valentín G. Baonza, Mercedes Taravillo, Álvaro Lobato

**Affiliations:** ^1^ MALTA‐Consolider Team and Departamento de Química Física Universidad Complutense de Madrid Plz. Ciencias 2 Madrid E‐28040 Spain; ^2^ Institut de Química Computacional i Catàlisi (IQCC) and Departament de Química Universitat de Girona, Campus de Montilivi Parc UdG Catalonia Girona E–17003 Spain; ^3^ ICREA Pg. Lluís Companys 23 Barcelona 08010 Spain; ^4^ Laboratorio de Difracción de Rayos X de Monocristal Servicio Interdepartamental de Investigación, Universidad Autónoma de Madrid Madrid E‐28049 Spain; ^5^ MatMoPol Research Group, Departamento de Química Inorgánica Universidad Complutense de Madrid Plz. Ciencias 2 Madrid E‐28040 Spain

**Keywords:** bidentate ligands, diruthenium, electronic structure, metal–metal bond, paddlewheel

## Abstract

Previous studies on bimetallic paddlewheel compounds have established a direct correlation between metal–metal distance and ligand torsion angles, leading to the rule that higher torsion results in longer metal‐metal bond distances. Here, the new discovery based on diarylformamidinate Ru₂⁵⁺ paddlewheel compounds [Ru_2_Cl(DArF)_4_] that show an opposite behavior is reported: higher torsions lead to shorter metal–metal distances. This discovery challenges the assumption that internal rotation solely impacts the δ bond. By combining experimental and theoretical techniques, it is demostrated that this trend is associated with previously overlooked π metal‐ligand interactions. These π metal‐ligand interactions are a direct consequence of the paddlewheel structure and the conjugated nature of the bidentate ligands. This findings offer far‐reaching insights into the influence of equatorial ligands and their π‐conjugation characteristics on the electronic properties of paddlewheel complexes. That this effect is not exclusive of diruthenium compounds but also occurs in other bimetallic cores such as ditungsten or dirhodium is demonstrated, and with other ligands showing allyl type conjugation. These results provide a novel approach for fine‐tuning the properties of these compounds with significant implications for materials design.

## Graphical abstract

Recent research challenges established notions in bimetallic paddlewheel compounds. Contrary to prior findings, higher torsion results in shorter metal–metal distances, not longer. This anomaly, attributed to overlooked π metal‐ligand interactions, highlights the importance of the π conjugated nature of the equatorial ligands in the electronic structure of metal–metal compounds, and offers new avenues for precise design of bimetallic compounds.

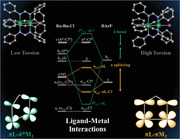

## 1 Introduction

Challenging accepted chemical rules has always been a fertile field to create new chemistry. Through these deliberate departures from convention, innovative insights emerge and researchers have uncovered new chemical avenues, as the discovery of noble gases compounds,^[^ ^1^ ^]^ bond orders higher than those predicted by p‐block elements,^[^ ^2^, ^3^ ^]^ or the formation of lanthanide metal‐to‐metal bonds with strong magnetic coupling,^[^ ^4^, ^5^, ^6^ ^]^ among others.

A widely accepted rule in metal‐metal complexes dictates that internal rotation increases the metal‐to‐metal distance.^[^ ^7^, ^8^ ^]^ This rule is considered essential for tailoring the properties of binuclear complexes. The manipulation of M–M distances and torsion angles has yielded valuable insights into electronic structure of metal–metal bonds,^[^ ^9^, ^10^, ^11^, ^12^ ^]^ encouraging applications in catalysis^[^ ^13^ ^]^ and materials science.^[^ ^14^, ^15^ ^]^

Based on extensive experimental observations^[^ ^16^, ^17^ ^]^ and comprehensive reviews in the literature,^[^ ^18^ ^]^ the torsion angle rule is generally regarded as applicable to all bimetallic compounds.^[^ ^7^, ^19^ ^]^ Its wide‐spread applicability is based on the underlying principles: as σ and π bonds remain essentially unaltered with increasing torsion angle, the lowering of δ orbital overlap weakens the M–M bond.^[^ ^7^ ^]^ Consequently, as the torsion angle approaches the staggered conformation, the metal–metal distance elongates.

In this work, we present a series of [Ru_2_Cl(DArF)_4_] (DArFˉ = diarylformamidinate) paddlewheel compounds that do *not* follow this pattern. Our results challenge the notion that internal rotation only affects the δ bond, and evidence the importance of the usually overlooked π metal‐ligand interactions in manipulating the electronic structure and properties of paddlewheel complexes.

Our research was motivated by the interesting case of the [Ru_2_(TiPB)_4_] (TiPB^−^ = 2,4,6‐triisopropyl benzoate) paddlewheel compound.^[^ ^20^ ^]^ This species presents a structure with a rotation angle of 11.9 – 15.2° and an unusually short Ru─Ru bond distance in comparison to others from the same family but with eclipsed conformations. This unexpected effect was also confirmed by DFT calculations performed for [Ru_2_(O_2_CH)_4_] and its Ru_2_^5+^ analogue, revealing that it takes places regardless of the oxidation state.^[^ ^21^ ^]^

Previous explanations for short metal‐metal distances based on steric *pressure* ^[^ ^22^, ^23^ ^]^ and bite angles^[^ ^24^ ^]^ of bidentate ligands do not suffice in this case. In the [Ru_2_(O_2_CH)_4_] species, the HCO_2_ˉ ligands are too small to produce a steric clash, therefore, negating the so‐called *wind up‐wind down* effect.^[^ ^22^, ^23^ ^]^

Given the complexity of the interactions involved in the δ‐bond formation, additional electronic effects derived from the interactions between the π‐systems of the equatorial bidentate ligands and the bimetallic core may be responsible for the unexpected variation of the metal‐metal distances observed in the mentioned paddlewheel complexes. Confirmation of this finding is of utmost importance in coordination chemistry, as this type of bidentate ligands are among the most widely used ligands.^[^ ^25^, ^26^ ^]^

The paddlewheel structure is the main architecture of bimetallic complexes. Most of the compounds synthesized and used to explore the exceptional properties of bimetallic complexes are based on this type of structure. This geometry allows a wide range of applications^[^ ^27^, ^28^ ^]^ thanks to the possibility of controlling their different coordination positions. In fact, these complexes have proven to be invaluable models for delving into the electronic structure^[^ ^2^, ^29^, ^30^ ^]^ and reactivity^[^ ^31^, ^32^, ^33^ ^]^ within the realm of coordination chemistry. However, while the effect of the axial ligands in paddlewheel complexes has been widely studied,^[^ ^34^, ^35^, ^36^, ^37^ ^]^ interactions through equatorial bidentate ligands remain quite unexplored.^[^ ^38^, ^39^ ^]^ Despite going relatively overlooked, understanding these equatorial interactions may offer an effective means to fine‐tune the electronic behavior and properties of such complexes. For instance, previous studies focusing on changing the nature of equatorial bidentate ligands show a strong effect on the magnetism,^[^ ^40^ ^]^ stabilized unusual metal‐metal bonds,^[^ ^41^ ^]^ or improved the reactivity of such complexes.^[^ ^42^, ^43^ ^]^

Here we present a series of Ru_2_^5+^compounds that defies the torsion angle rule. Optical absorption measurements and DFT calculations are used to provide strong evidence of the metal‐ligand interactions and their complex nature. We conclude demonstrating the implications of our work by explaining the torsion‐distance variation for different bimetallic complexes. In fact, this effect is observed not only for the compounds of study and the previously described [Ru_2_(O_2_CR)_4_]^0,+^ species,^[^ ^20^, ^21^ ^]^ but also for several paddlewheel complexes bearing different transition metal ions and bidentate ligands. These results highlight the generality of the unrevealed torsion effect in these species, and the potential benefits derived from modulating the M_2_‐πLigand interactions to fine‐tune their properties.

## 2 Results and Discussion

**Figure 1:**
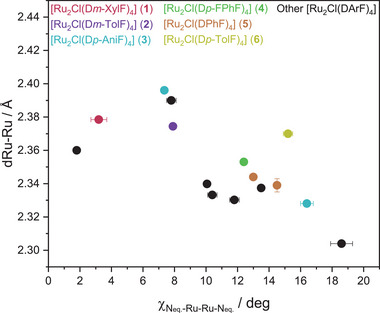
Variation of Ru‐Ru bond distances with the N_eq.‐_Ru‐Ru‐N_eq_. dihedral angle (χ_Neq.‐Ru‐Ru‐Neq._). Filled dots correspond to the synthesized compounds 1 (red), 2 (purple), 3 (blue), 4 (green), 5 (orange) and 6 (yellow) and black circles correspond to other bibliographic data. Standard deviations in the determination of torsion angles and Ru‐Ru distances for each compound are included as bars. Data and their associated standard deviations are collected in Table S4 (Supporting Information).

**Scheme 1:**
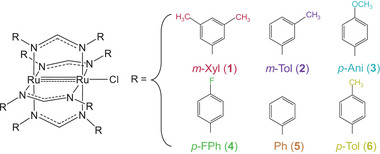
Generic representation of the [Ru_2_Cl(DArF)_4_] paddlewheel structure along with the DArFˉ substituent groups of the synthesized compounds 1–6.

We first recap in **Figure** **1** a comprehensive summary of our findings. We opted for a series of [Ru_2_Cl(DArF)_4_] complexes as test‐bed models for our analysis (see structure in **Scheme** **1**). Our preference for the DArFˉ ligand was due to its ability to exhibit a wide range of equatorial‐metal torsion angles, from almost 0.00°^[^ ^44^ ^]^ to 27.38°, the maximum observed in paddlewheel compounds with different metal centers.^[^ ^45^ ^]^ This approach allows us to study the influence of the internal rotation on the metal‐metal distance without changing the nature of the ligand.^[^ ^16^, ^17^ ^]^

Unfortunately, [Ru_2_Cl(DArF)_4_] compounds with torsion angles below 10° are scarce and, to the best of our knowledge, only two such compounds have been reported in the literature.^[^ ^35^, ^46^, ^47^ ^]^ To address this gap, we ventured into the synthesis of new compounds. Our protocols were guided by previously reported crystal structures, where ligands with aromatic rings that presented substituents in *meta* positions tend to exhibit smaller twisting angles. Finally, we succeeded in synthesizing [Ru_2_₂Cl(D*m*‐XylF)₄] (1) and [Ru₂Cl(D*m*‐TolF)₄] (2), which present N‐Ru‐Ru‐N dihedral angles of 3.23° and 7.89°, respectively.

In addition to 1 and 2, we further opted to synthesize [Ru₂Cl(D*p*‐AniF)₄] (3), [Ru₂Cl(D*p*‐FPhF)₄] (4), [Ru₂Cl(DPhF)₄] (5), and [Ru₂Cl(D*p*‐TolF)₄] (6) (see Scheme 1). This selection allowed us to perform magnetic (SQUID) and diffuse reflectance measurements over a broad range of torsion angles. The detailed synthetic procedures and characterization of the compounds by mass spectrometry (Figures S1–S6, Supporting Information) and single crystal X‐ray diffraction analysis of compounds 1–3 can be found in the Supporting Information file (Figures S7–S9 and Tables S1–S5, Supporting Information).

Despite the scatter observed in the metal‐metal distances of Figure 1, an obvious trend is revealed: compounds with higher torsion angles display shorter Ru‐Ru distances, in clear contrast to the torsion angle rule. Among other subtle contributions that will be analyzed later, differences in the equatorial ligands' donor capacity^[^ ^18^ ^]^ and slight variations of the Ru‐Cl distances,^[^ ^35^ ^]^ seem to be responsible for the observed dispersion with respect to a smooth variation of the distances versus the torsion angle.

In any case, the most revealing confirmation of the observed trend in the Ru‐Ru distances is provided by the results of those compounds showing several polymorphs due to various crystallization conditions. For example, compound 3 has three crystal structures, two documented in the literature,^[^ ^35^, ^48^ ^]^ and an additional one obtained in this work. In these structures, our measured Ru‐Ru bond distances 2.396, 2.390, and 2.328 Å strongly correlate with torsion angles of 7.36°, 7.81° and 16.39°, respectively. Similarly, 5 exhibits a 2.344 Å bond distance at 12.99°, which shortens to 2.339 Å at a torsion angle of 14.53°.

Previous studies^[^ ^16^, ^17^, ^18^ ^]^ discussing the torsion angle rule have emphasized the significance of analyzing data from bimetallic units with identical oxidation states, equivalent electronic configurations, and comparable steric effects. This approach ensures that any observed variations in metal─metal bond lengths primarily result from torsional effects. To rule out the potential influence of these effects on the Ru‐Ru trend, we have rigorously screened them within our dataset.

All the compounds featured in Figure 1 present an analogous paddlewheel structure, with four bidentate DArFˉ ligands equatorially coordinated, and an additional chloride at one of the axial positions. This geometry guarantees that all the compounds have the same formal oxidation state (5+) in the bimetallic moiety. Moreover, the Ru‐Ru‐Cl angle remains nearly 180° in all cases (see data in Table S4, Supporting Information), preventing second‐order Jahn‐Teller effects^[^ ^49^, ^50^ ^]^ which could otherwise modify the observed metal‐metal distances.

Likewise, it is well‐established that Ru‐Ru bond distances in [Ru_2_Cl(DArF)_4_] species experience significant elongation in the low‐spin configuration.^[^ ^27^, ^35^, ^47^ ^]^ This elongation is believed to arise from the presence of one additional electron in the π* orbitals, shifting the electronic configuration from σ^2^π⁴δ^2^(π*δ*)^3^ to σ^2^π⁴δ^2^π*^3^. To investigate this effect, we performed temperature dependent magnetic susceptibility measurements for all the synthesized compounds 1–6. SQUID measurements (Figures S10–S15, Supporting Information) revealed that compounds 2, 4, 5, and 6 exhibit a high‐spin (*S* = 3/2) configuration, while compounds 1 and 3 exhibit an intermediate configuration between high‐spin and low‐spin at room temperature. Even in these unusual cases, a substantial electron population occupies the δ* orbitals, suggesting that changes in the electronic configuration alone cannot explain the observed variations in Ru─Ru bond distances.

Changes in the bite angle and steric pressure of equatorial ligands could also lead to shorter metal‐metal distances,^[^ ^22^, ^51^, ^52^ ^]^ offering a potential explanation for the trend observed in the Ru₂⁵⁺ paddlewheel compounds. The effect of the biting angle is negligible for the Ru_2_^5+^ species studied here, similar to previous observations in [Ru_2_(DArF)_4_]^[^ ^53^ ^]^ and [Mo_2_(DArF)_4_]^[^ ^54^ ^]^ complexes. In fact, the N‐C‐N angles in our set of compounds barely change, ranging from 119.05° to 123.48° (Table S5, Supporting Information). Furthermore, the changes in the distance between the nitrogen binding atoms, which ultimately drive the reduction in Ru‐Ru length, are less than 0.02 Å (Table S5, Supporting Information). Finally, changes in the N‐Ru‐Ru angle due to steric pressure effects are marginal, typically less than two degrees (Table S5, Supporting Information), unambiguously demonstrating the regularity among the studied compounds in terms of steric hindrance.

Since traditional explanations rooted in changes to oxidation states, electronic configuration, and steric pressure fail to account for the observed variation in Ru─Ru bond length, why does bond length shortening occur in paddlewheel Ru_2_^5+^ compounds upon ligand twisting?

**Figure 2:**
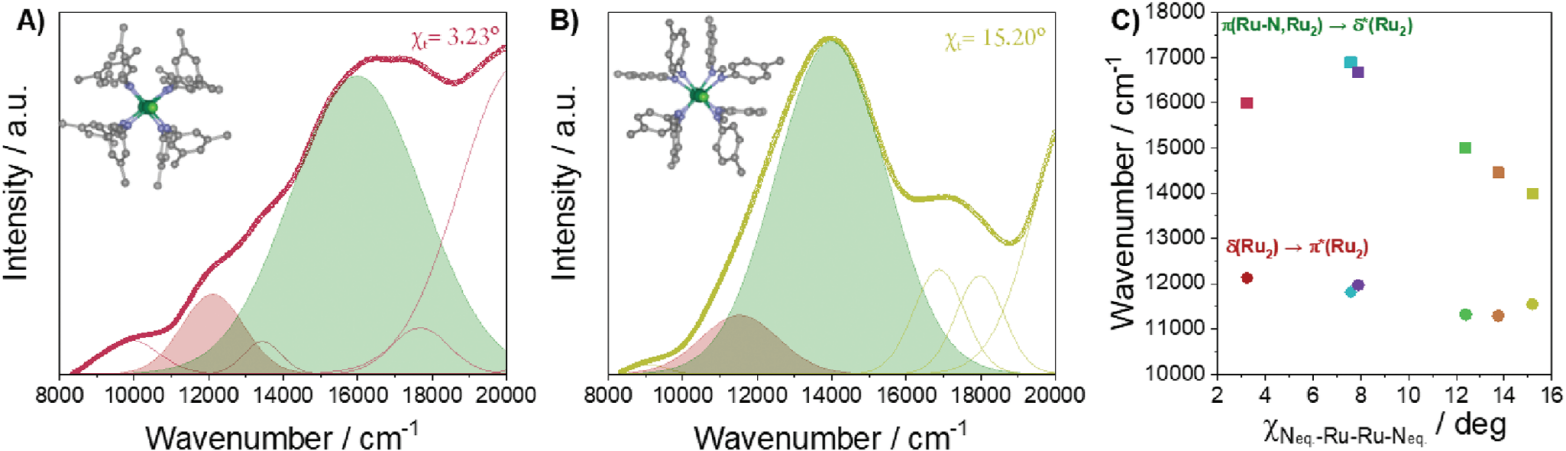
Representation of the structure through the Ru‐Ru‐Cl axis and diffuse reflectance spectra of compounds A) 1 (red) and B) 6 (yellow). δ(Ru_2_) → π*(Ru_2_) and π(Ru‐N, Ru_2_) → δ*(Ru_2_) transitions are represented by red and green shaded curves, respectively. C) Variation of the band centre of π(Ru‐N, Ru_2_) → δ*(Ru_2_) (squares) and δ(Ru_2_) → π*(Ru_2_) (circles) electronic transitions with the N_eq._‐Ru‐Ru‐N_eq._ dihedral angle for compounds 1 (red), 2 (purple), 3 (blue), 4 (green), 5 (orange) and 6 (yellow). In compound 5, the torsion angle is the average of the two bibliographic data (see ESI file).

The analysis of the electronic spectra provides a first insight. When we examine the diffuse reflectance spectra of compounds characterized by low (1, 3.23°) and high (6, 15.20°) torsion angles, the main absorption peak shifts to lower energies, as illustrated in **Figure** **2**A,B. To a first approximation, one may assume that this difference is due to changes in the magnetic properties. However, this rationale does not explain why the spectra of 2 and 3 are similar to each other (see Figures S17 and S18, Supporting Information), despite their different spin states but just analogous torsion angles.

A more detailed analysis clarifies that these spectral shifts arise from two distinct bands (see Figures S16 to S21, Supporting Information). We have assigned these bands to the δ(Ru_2_) → π*(Ru_2_) and π(Ru‐N,Ru_2_) → δ*(Ru_2_) transitions, based on previous observations in [Ru_2_Cl(ap)_4_] (ap = anilinopyridinate) species,^[^ ^55^ ^]^ in which the NCN bidentate ligand is closely related to DArFˉ. UV‐Vis, and magnetic circular dichroism (MCD) spectroscopies, which unequivocally identify d‐d metal transitions, along with DFT calculations, placed these transitions at ≈10 000 and 13 500 cm⁻¹,^[^ ^55^ ^]^ respectively, and our experimental results closely fit with these values.

It is worth noting that in previous studies of [Ru₂Cl(DArF)₄] compounds in dichloromethane, these bands were assigned to the δ(Ru_2_) → π*(Ru_2_) and π*(Ru_2_) → σ*(Ru‐N) transitions occurring at 15 000 and 17 000 cm⁻¹.^[^ ^56^ ^]^ Nevertheless, this assignment has been a subject of debate, since it initially relied on studies involving acetate compounds.

In fact, acetate compounds exhibit an inversion of the π* and δ* orbitals compared to the diarylformamidinates,^[^ ^57^ ^]^ thus indicating significant differences in the electronic structure of these compounds. In any case, we have ruled out the latter assignment since a d‐d parity forbidden metal‐to‐metal δ(Ru_2_) → π*(Ru_2_) transition rather than the dipole‐allowed charge transfer π(Ru‐N, Ru_2_) → δ*(Ru_2_) transition should be associated to the most intense band in our spectra.

In Figure 2C, we show the evolution of the π(Ru‐N,Ru_2_) → δ*(Ru_2_) and δ(Ru_2_) → π*(Ru_2_) bands as a function of the torsion angle for compounds 1 to 6. The δ(Ru_2_) → π*(Ru_2_) band exhibits a decrease in energy as the torsional twisting increases. This conforms to accepted expectations because the delta bond weakens under torsion. However, it is noteworthy that the π(Ru‐N,Ru_2_) → δ*(Ru_2_) transition slightly increases and suddenly drops at high torsion angles with a decrease four times more pronounced than the δ*(Ru_2_) → π*(Ru_2_) one. Such a slope change should not be solely attributed to delta bond effects, since energy variations upon torsion in the δ and δ* orbitals should be similar.^[^ ^58^ ^]^ Therefore, according to the previous discussion, the π orbital must be undergoing an energy increase, and this provides evidence that changes in the electronic structure may underlie the unique torsion effects in paddlewheel complexes. This observation is quite remarkable, as per traditional notions of the metal‐metal bonding, internal rotation should not impact the M‐M molecular structure beyond the delta bond.^[^ ^7^ ^]^

From a chemical perspective, the primary distinction between paddlewheel and M₂X₈ complexes (M = Mo, Cr, X = Cl) that adhere to the torsion angle rule is the nature of the equatorial ligands. DArF^−^ ligands are known to exhibit a π conjugation pattern resembling allyl‐type anions,^[^ ^26^, ^59^, ^60^ ^]^ which can interact with the metal‐metal framework.^[^ ^41^, ^61^, ^62^ ^]^ This suggests that equatorial ligands play an additional role in affecting the electronic structure of the metal‐metal core upon twisting, a situation which has not been considered before to the best of our knowledge.

To further analyze the potential effect of the ligands in the metal manifold, we have carried out DFT calculations (at S12g/TZ2P, see Supporting Information for further details) at different torsion angles for compound 6 as a model system. Our calculations nicely reproduce the behavior observed in our analysis of the crystallographic data. The Ru‐Ru bond length decreases as the torsion angle increases, which substantiates the validity of our computations (see Figure S22, Supporting Information). The validity of compound 6 as test‐bed model for the computational study is confirmed after performing equivalent DFT calculations for compound 4, for which analogous results regarding the internal rotation effect on the Ru‐Ru bond distance were obtained (Figure S25, Supporting Information).

**Figure 3:**
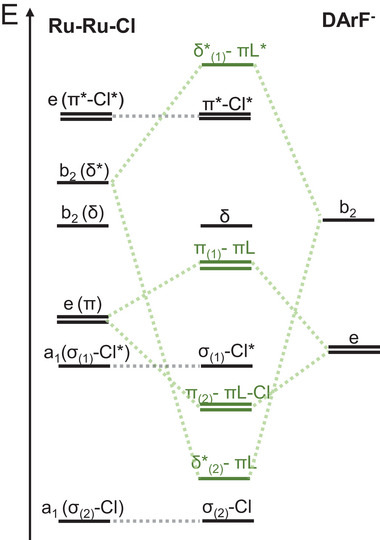
Schematic molecular orbital diagram showing the interactions between the π NCN orbitals of the DArF^−^ with the metal‐metal manifold obtained from the DFT calculations in [Ru_2_Cl(D*p*‐TolF)_4_] (6).

We show in **Figure** **3** a schematic molecular orbital (MO) diagram focusing on the symmetry‐matched interactions between the diruthenium core and the π conjugated N‐C‐N orbitals of the equatorial ligands in the absence of distortion. While σ, δ and π* orbitals of the Ru_2_ fragment remain unaffected, the π and δ* exhibit strong interactions with the *e* and *b_2_* π‐orbitals of the DArF^−^, respectively.

In the case of the δ* orbital, the strong ligand interaction results in bonding and antibonding *b*_2_ molecular orbitals. The bonding component δ*_(2)_‐πL is close to the σ_(2)_‐Cl molecular orbital. Conversely, the antibonding orbital (δ*_(1)_‐πL*) places at higher energies than π*, which explains the observed orbital inversion in [Ru₂Cl(DArF)₄] species compared to [Ru₂Cl(OCR)₄] acetate compounds.^[^ ^63^ ^]^

Notably, a similar situation occurs for the π(Ru_2_) orbitals. Their overlap with the ligands leads to the emergence of two new molecular orbitals, denoted as π_(1)_‐πL and π_(2)_‐πL‐Cl which correspond to the higher and lower energy combinations, respectively. While interactions between ligands and low energy π orbitals are known to occur primarily through axial positions,^[^ ^35^, ^36^ ^]^ in the case of the equatorial sites they are not usually taken into account. Although theoretical predictions of πRu_2_‐πL equatorial interactions for [Pd_2_(HNCHNH)_4_],^[^ ^41^ ^]^ [Fe_2_(DArF)_4_]^[^ ^61^ ^]^ and [M_2_Cl_n_(hpp)_4_] (M = Mo, W; n = 0, 2; hppˉ = anion from deprotonation of 1,3,4,6,7,8‐hexahydro‐2*H*‐pyrimido[1,2‐*a*]pyrimidine)^[^ ^64^ ^]^ compounds were found in the literature, their effect on the metal‐metal framework upon torsion have remained unexplored until now.

**Figure 4:**
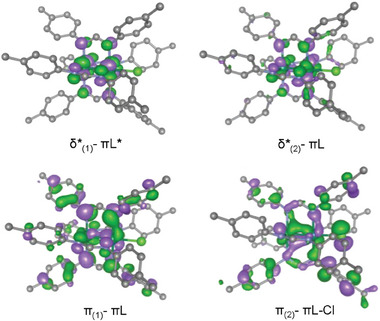
Molecular orbitals isosurfaces for the different δ*‐πL and π‐πL orbitals of [Ru_2_Cl(D*p*‐TolF)_4_] (6) at an iso‐value of 0.04. Hydrogen atoms have been omitted for clarity.

The features of the δ*‐πL and π‐πL orbitals reveal their impact on the bimetallic unit upon torsion (**Figure** **4**). In the case of the δ*‐πL (*b_2_*) molecular orbitals, their mixing with the ligands must be minimally affected by rotation, given that the δ* orbitals lie parallel to the N p orbitals. This contrasts to the π‐πL orbitals, where a strong dependence upon twisting is expected. Here, some of the p orbitals of the N atoms lie perpendicular to the metal ones, which can alter the metal‐ligand overlap due to the tilting of N p orbitals caused by torsion.

**Figure 5:**
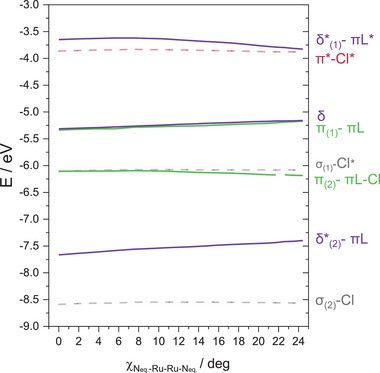
Changes in the energies of the beta molecular orbitals in [Ru_2_Cl(D*p*‐TolF)_4_] at various torsion angles. Purple solid lines: δ_(1)_*‐πL* and δ_(2)_*‐πL, green solid lines: π_(1)_‐πL and π_(2)_‐πL, black dashed line: σ_(2)_‐Cl, σ_(1)_‐Cl* and red dashed line: π*.

**Figure** **5** shows how the MO diagram evolves with the torsion angle for compound 6. Between 0 and 9 degrees the energy of the π_(1)_‐πL and π_(2)_‐πL‐Cl orbitals slightly change, as from we observe a substantial enhancement of the π‐πL mixing with the internal rotation in agreement with the anticipated analysis of MO orbital features. This mixing enhancement translates into an increase of the energy gap between π_(1)_‐πL and π_(2)_‐πL‐Cl of 0.24 eV upon distortion (see Figure S23, Supporting Information). This is a key result in our analysis, because it perfectly agrees with our findings in the electronic spectra, where we concluded that π(Ru‐N, Ru_2_) orbitals should also be changing their energy upon changing the torsion angle.

The δ*‐πL interaction also changes its energy at different rates upon twisting, but in this case, it weakens when increasing the torsion angle at values higher than 9 degrees (see Figure S23, Supporting Information). This effect is only marginally related to the ligands. Instead, it is a consequence of the decreasing antibonding interactions between the d‐d orbitals involved in the delta bond. Indeed, there is also a 0.17 eV increase in energy of the δ orbital, where no N binding atom contributions are observed (see Figure S24, Supporting Information), which is consistent with a purely overlap decrease.

To discard any contribution of the aromatic substituent in the torsion effect, the relative energies of the molecular orbitals, as well as the π‐πL and δ*‐πL splitting's for compounds 4 and 6, are compared at two different geometries (torsion angles 8 and 15°, approximately). The data are shown in Table S8 (Supporting Information). For both species, the π‐πL splitting increases ≈0.05 eV in this torsion range, while δ*‐πL splitting decreases ≈0.1 eV. These changes in the Ru_2_‐πL interactions are equivalent in 4 and 6, and higher than the energy differences between both complexes, indicating that, for practical purposes, the torsion angle effect is analogous regardless the DArF^−^ substituent.

The different variation of the π metal‐ligand mixing and the δ bond reveals the origin of the unique variation of the Ru‐Ru distances in [Ru₂Cl(DArF)₄] paddlewheel compounds. There is a tug‐of‐war for the stabilization of metal‐metal bonds in these species, and torsion effects reveal this phenomenon.

**Figure 6:**
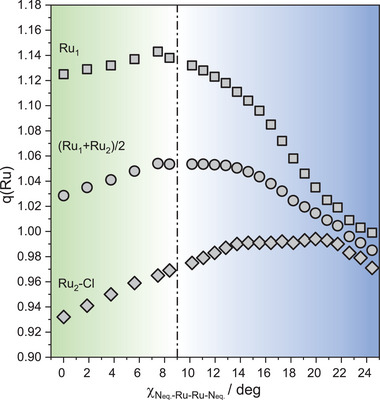
Mulliken charge distribution calculated for compound [Ru_2_Cl(Dp‐TolF)_4_] (6) at various torsion angles. The charges calculated for the ruthenium ion free of axial interactions (Ru_1_) are represented by squares, while rhombi represent the ruthenium ion linked to the chloride ligand (Ru_2_‐Cl) and circles the average between both.

At high torsion angles the δ bond weakens due to an overlap decrease, which should result in a metal‐metal distance elongation as previously observed in M_2_X_8_ compounds. This effect is opposite and exceeded by the increase of π metal‐ligand mixing, where a substantial charge transfer from the ligands to the bimetal core is produced. This charge transfer is confirmed by a Mulliken charge analysis in compound 6 (see **Figure** **6**). Despite the differences in the environment of both Ru ions giving rise to different trends in charges for Ru_1_ and Ru_2_‐Cl, the global effect is that the average charge fades upon twisting from ≈9 degrees. Such a charge transfer diminishes the electrostatic repulsions between the metallic atoms resulting in an expansion of the d orbitals. Consequently, a more effective π d‐d overlap is produced, reinforcing the metal‐metal bond, and thus decreasing the metal‐metal distance. This situation differs from the low torsion regime (below 9°), where the δ*‐πL and π‐ πL orbitals slightly change. In this case, both the δ bond weaking and the π‐πL mixing are of the same magnitude resulting in a minor charge increase and evidencing only a slight distance decrease with torsion.

In other words, in Ru_2_^5+^ paddlewheel complexes, the charge transfer induced by the π metal‐ligand mixing overcomes the delta bond weakening, resulting in a decrease in the Ru‐Ru distances upon high twisting. The importance of charge effects in the bimetallic unit has been noted in the literature before,^[^ ^7^, ^65^, ^67^ ^]^ and in some way the ligand mixing effect on the metal orbitals we have just described is similar to the one produced upon reduction. For instance, in the case of [Tc_2_Cl_8_]^nˉ^ (n = 2, 3), shorter Tc‐Tc distances are found for [Tc_2_Cl_8_]^3^ˉ (bond order 3.5) than for [Tc_2_Cl_8_]^2^ˉ (bond order 4). This effect was attributed, as in our case, to a more effective overlap of the d orbitals due to the charge decrease of the technetium ions from +3 in [Tc_2_Cl_8_]^2^ˉ to +2.5 in [Tc_2_Cl_8_]^3^ˉ.^[^ ^68^ ^]^

The fact that a subtle energy change in π interactions affects the electronic structure in paddlewheel bimetallic species is a particularly striking observation. In fact, the influence of bidentate equatorial ligands has traditionally been discussed in terms of sigma interactions and their relationship with the donor capacity.^[^ ^69^, ^70^, ^71^ ^]^ Therefore, to further test whether the ligand effects are unique to Ru_2_^5+^ compounds, or whether they are a general feature of the paddlewheel structure, we have preliminarily examined other metal‐metal compounds.

**Figure 7:**
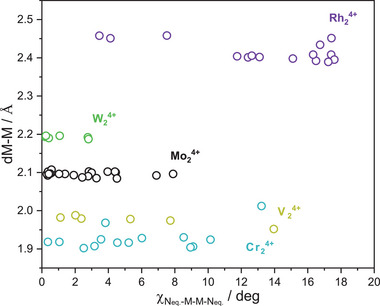
Changes in metal‐to‐metal bond distances with the twisting N_eq._‐M‐M‐N_eq._ angle for DArFˉ paddlewheel complexes of Cr_2_^4+^ (blue), V_2_^4+^ (yellow), Mo_2_^4+^ (black), W_2_^4+^ (green) and Rh_2_^4+^ (purple).

In **Figure** **7**, we present a comparative analysis of metal‐to‐metal bond distances in various DArFˉ paddlewheel complexes, including Cr_2_⁴⁺, V_2_⁴⁺, Mo_2_⁴⁺, W_2_⁴⁺ and Rh_2_⁴⁺. Data are collected from crystal structures reported in the literature (Tables S9–S13, Supporting Information).

Remarkably, Rh_2_^4+^ and V_2_^4+^ species exhibit the same effect as Ru_2_^5+^ compounds. They both decrease their M─M bond distance as a function of the torsion angle. Since delta bond contribution barely exists in these compounds (their electronic configurations are σ^2^π^4^δ^2^δ*^2^π*^4^ and σ^2^π^4^, respectively), the π metal‐ligand mixing must be wholly responsible for the observed variation in the distance.

Further insight into the nature of π metal‐ligand interaction can be gained by analyzing the Cr_2_^4+^, Mo_2_^4+^ and W_2_^4+^ species. Although these compounds present the same electronic configuration,^[^ ^72^ ^]^ σ^2^π^4^δ^2^, with a clear contribution of δ overlap, their metal‐to‐metal distances show quite different patterns. Cr─Cr bonds clearly elongate upon twisting, but Mo‐Mo and W‐W barely change. Being lower in the periodic group, the effective charges of Mo and W are smaller and, therefore, their d orbitals are less contracted, leading to a more effective orbital mixing with the ligands. As the mixing increases, the contribution of the ligand becomes more apparent, changing the trend in the distances and torsion angles. This observation demonstrates that the predominance of the π metal‐ligand interactions over the δ bonding depends on the relative energies of the d‐metal and π‐NCN orbitals.

As happened with the different bimetallic units, the effects we have described above are expected to occur in other paddlewheel compounds. The effect of metal‐ligand interactions is not exclusive to the ligands we have selected. In fact, they are a consequence of the π‐conjugation nature of the equatorial ligand's atoms involved in the bridging. Guanidinates, acetates, triazenides or anilinopyridinates (ap^−^) are good candidates to display this effect since all exhibit an allyl type conjugation like DArFˉ ligands. We have traced the literature for [Ru_2_Cl(ap)_4_] and [Rh_2_Cl(ap)_4_] analogues and found that they also exhibit a π‐ligand metal mixing, since the metal‐metal bond shortens upon twisting (Tables S14 and S15, Figure S26, Supporting Information). The presence of π metal‐ligand interactions in paddlewheel bimetallic complexes is thus more common than one previously anticipates from the torsion angle rule. These interactions are not exclusive to a specific metal unit or ligand, highlighting their generality. This may open a new field in coordination chemistry, as the modulation of these interactions by a targeted combination of metal ions and bidentate ligands can fine‐tune the electronic properties of paddlewheel compounds.

The capability to modify metal‐metal distances and charges through π interactions with the ligands stimulate analogies with models based on oxidation and reduction in altering catalytic properties,^[^ ^73^ ^]^ stabilize unusual electronic configurations^[^ ^41^ ^]^ or producing short metal‐metal distances.^[^ ^2^ ^]^ These are just a few examples, but we firmly believe that understanding how metal‐ligand interactions shape the structural and electronic properties of metal‐metal paddlewheel compounds offers a new perspective for future research in this field.

## 3 Conclusion

We have conducted an extensive investigation into the behavior of metal‐metal distances in [Ru_2_Cl(DArF)_4_] compounds. Contrary to the established paradigm in metal‐metal complexes, we have shown that torsion angles provoke a shortening in the metal‐metal bond distance in these species.

Our results reveal that overlooked metal‐equatorial ligand interactions are the key factor responsible for the anomalous behavior of Ru_2_⁵⁺ paddlewheel complexes. A comprehensive array of techniques, including single crystal X‐ray diffraction, DFT calculations and optical absorption measurements, clearly reveal that these interactions predominantly involve the low energy π orbitals of the metal core.

This discovery defies the established ideas suggesting that internal rotation only affects the delta bond. When bidentate bridging ligands based on allyl type conjugation are present, not only does the delta bond play a role upon torsion, the π metal‐ligand interactions also contribute by diminishing the positive charge on the diruthenium unit and thus improving the π metal‐metal overlap.

While π metal‐ligand interactions are known to occur through the axial position, they have not been usually considered in the case of equatorial ones. However, according to our results involving various bimetallic units and ligands, these interactions are more common than previously anticipated. Many different ligands such as guanidinates, acetates, triazenides or anilinopyridinates are proposed to exhibit this effect, since the only requisite is that they present a π‐conjugation pattern in a paddlewheel structure.

These equatorial effects are dependent on the nature of the metal ions, and they can be easily switched depending on the relative energies of the metal and the π ligand orbitals. We believe that this research opens exciting opportunities for targeted design and modification of metal complexes. The possibilities include customizing their electronic properties, enhancing their reactivity, and fine‐tuning their applications in catalysis, materials science, and coordination chemistry.

## Conflict of Interest

The authors declare no conflict of interest.

## Supporting information

Supporting Information

## Data Availability

The data that support the findings of this study are openly available in IOCHEM‐BD platform at [https://doi.org/10.19061/iochem‐bd‐4‐68], reference number 1.
