# Supplementary material for: Torsion Effects Beyond the δ Bond and the Role of π Metal‐Ligand Interactions
Source: Adv Sci (Weinh). 2024 Apr 3;11(25):2401293. doi: 10.1002/advs.202401293 (PMC11220682; doi:10.1002/advs.202401293)
Supplement: Supplementary file 1 — Supporting Information [file ADVS-11-2401293-s001.pdf]

ADVANCED SCIENCE
Open Access

Supporting Information

for *Adv. Sci.*, DOI 10.1002/advs.202401293

Torsion Effects Beyond the δ Bond and the Role of π Metal-Ligand Interactions

*Almudena Inchausti, Rosa Mollfulleda, Marcel Swart, Josefina Perles, Santiago Herrero, Valentín G. Baonza, Mercedes Taravillo and Álvaro Lobato**

Supplementary Information

Torsion effects beyond the δ bond and the role of π metal-ligand interactions

*Almudena Inchausti,[a] Rosa Mollfulleda,[b] Marcel Swart,[b],[c] Josefina Perles,[d] Santiago Herrero,[e] Valentín García-Baonza,[a] Mercedes Taravillo[a] and Álvaro Lobato[a],**

[a] A. Inchausti, V. G. Baonza, M. Taravillo, A. Lobato*
MALTA-Consolider Team and Departamento de Química Física, E-28040 Madrid, Spain
Universidad Complutense de Madrid
Plz. Ciencias 2, E-28040 Madrid, Spain
E-mail: a.lobato@ucm.es

[b] R. Mollfulleda, M. Swart
Institut de Química Computacional i Catàlisi (IQCC) and Departament de Química
Universitat de Girona
Campus de Montilivi, Parc UdG, Girona E–17003 Catalonia, Girona, Spain

[c] M. Swart
ICREA
Pg. Lluís Companys 23, 08010 Barcelona, Spain

[d] J. Perles
Laboratorio de Difracción de Rayos X de Monocristal
Servicio Interdepartamental de Investigación
Universidad Autónoma de Madrid, E-28049 Madrid, Spain

[e] S. Herrero
MatMoPol Research Group. Departamento de Química Inorgánica
Universidad Complutense de Madrid
Plz. Ciencias 2, E-28040 Madrid, Spain

DOI: 10.1002/anie.2021XXXXX

S.1. EXPERIMENTAL SECTION 4
S.2. MASS SPECTROMETRY 6
Figure S1. Positive ion ESI mass spectrum of $[Ru_2Cl(D\textit{m}\text{-XylF})_4]$ (**1**) 6
Figure S2. Positive ion ESI mass spectrum of $[Ru_2Cl(D\textit{m}\text{-TolF})_4]$ (**2**).......................... 7
Figure S3. Positive ion ESI mass spectrum of $[Ru_2Cl(D\textit{p}\text{-AniF})_4]$ (**3**) 7
Figure S4. Positive ion ESI mass spectrum of $[Ru_2Cl(D\textit{p}\text{-FPhF}]_4$ (**4**)........................... 8
Figure S5. Positive ion ESI mass spectrum of $[Ru_2Cl(DPhF)_4]$ (**5**) 8
Figure S6. Positive ion ESI mass spectrum of $[Ru_2Cl(D\textit{p}\text{-TolF})_4]$ (**6**) 9
S.3. SINGLE CRYSTAL X-RAY DIFFRACTION.. 9

Figure S7. Asymmetric unit of **1·1.82H$_2$O**. Hydrogen atoms are omitted for clarity. Color code: dark green for Ru, light green for Cl, red for O, blue for N and grey for C. The ellipsoids are represented with a probability of 30%. 10

Table S1. Sample and crystal data for **1·1.82H$_2$O**... 10

Figure S8. Asymmetric unit of **2·0.5 H$_2$O.** Hydrogen atoms are omitted for clarity. Color code: dark green for Ru, light green for Cl, red for O, blue for N and grey for C. The ellipsoids are represented with a probability of 30%.. 12

Table S2. Sample and crystal data for **2·0.5H$_2$O**... 12

Figure S9. Asymmetric unit of **3·0.5CH$_2$Cl$_2$**. Hydrogen atoms are omitted for clarity. Color code: dark green for Ru, light green for Cl, red for O, blue for N and grey for C. The ellipsoids are represented with a probability of 30%. 14

Table S3. Sample and crystal data for **3·0.5CH$_2$Cl$_2$**. 14

S.4. M-M BOND DISTANCES AND TORSION ANGLES IN [Ru$_2$Cl(DArF)$_4$] COMPOUNDS 15

Table S4. Ru-Ru bond distances and N$_{eq.}$-Ru-Ru-N$_{eq.}$ dihedral angles for compounds with general formula [Ru$_2$Cl(DArF)$_4$]. 15

Table S5. NCN bite angles, distances between nitrogen binding atoms from the equatorial ligands, and N-Ru-Ru angles for compounds with general formula [Ru$_2$Cl(DArF)$_4$]. .. 15

S.5. MAGNETIC MEASUREMENTS 16

Figure S10. Temperature dependence of $\chi_M T$ for powder sample of [Ru$_2$Cl(D*m*-XylF)$_4$] (**1**) 16

Figure S11. Temperature dependence of $\chi_M T$ for powder sample of [Ru$_2$Cl(D*m*-TolF)$_4$] (**2**) 16

Figure S12. Temperature dependence of $\chi_M T$ for powder sample of [Ru$_2$Cl(D*p*-AniF)$_4$] (**3**) 17

Figure S13. Temperature dependence of $\chi_M T$ for powder sample of [Ru$_2$Cl(D*p*-FPhF)$_4$] (**4**) 17

Figure S14. Temperature dependence of $\chi_M T$ for powder sample of [Ru$_2$Cl(DPhF)$_4$] (**5**) 18

Figure S15. Temperature dependence of $\chi_M T$ for powder sample of [Ru$_2$Cl(D*p*-TolF)$_4$] (**6**) 18

S.6. DIFFUSE REFLECTANCE SPECTROSCOPY 18

Figure S16. The Gaussian fit of the diffuse reflectance spectrum of compound [Ru$_2$Cl(D*m*-XylF)$_4$] (**1**). The δ(Ru$_2$) → π*(Ru$_2$) and π(Ru-N,Ru$_2$) → δ*(Ru$_2$) electronic transitions are represented in red and green, respectively. 19

Figure S17. The Gaussian fit of the diffuse reflectance spectrum of compound [Ru$_2$Cl(D*m*-TolF)$_4$] (**2**). The δ(Ru$_2$) → π*(Ru$_2$) and π(Ru-N,Ru$_2$) → δ*(Ru$_2$) electronic transitions are represented in red and green, respectively. 19

Figure S18. The Gaussian fit of the diffuse reflectance spectrum of compound [Ru$_2$Cl(D*p*-AniF)$_4$] (**3**). The δ(Ru$_2$) → π*(Ru$_2$) and π(Ru-N,Ru$_2$) → δ*(Ru$_2$) electronic transitions are represented in red and green, respectively. 20

Figure S19. The Gaussian fit of the diffuse reflectance spectrum of compound [Ru_2Cl(D*p*-FPhF)$_4$] (**4**). The δ(Ru_2) → π*(Ru_2) and π(Ru-N,Ru_2) → δ*(Ru_2) electronic transitions are represented in red and green, respectively. 20

Figure S20. The Gaussian fit of the diffuse reflectance spectrum of compound [Ru_2Cl(DPhF)$_4$] (**5**). The δ(Ru_2) → π*(Ru_2) and π(Ru-N,Ru_2) → δ*(Ru_2) electronic transitions are represented in red and green, respectively. 21

Figure S21. The Gaussian fit of the diffuse reflectance spectrum of compound [Ru_2Cl(D*p*-TolF)$_4$] (**6**). The δ(Ru_2) → π*(Ru_2) and π(Ru-N,Ru_2) → δ*(Ru_2) electronic transitions are represented in red and green, respectively. 21

Table S6. Tentative assignment for the observed bands in diffuse reflectance spectra of compounds **1** – **6**, based on previous calculations performed on [Ru_2Cl(ap)$_4$] (ap = anilinopyridinate).[16] 21

S.7. DFT CALCULATIONS 22

Figure S22. Variation of Ru-Ru bond distances with the $N_{eq.}$-Ru-Ru-$N_{eq.}$ dihedral angle in compound [Ru_2Cl(D*p*-TolF)$_4$] (**6**). 23

Table S7. Ru-Ru bond distances and $N_{eq.}$-Ru-Ru-$N_{eq.}$ dihedral angles angle in compound [Ru_2Cl(D*p*-TolF)$_4$] (**6**). 23

Figure S23. Splitting of the A) π-πL and B) δ*-πL molecular orbitals at various angles calculated for [Ru_2Cl(D*p*-TolF)$_4$] (**6**). 24

Figure S24. Isosurface maps of the Ru-Ru based molecular orbitals of compound [Ru_2Cl(D*p*-TolF)$_4$] (**6**), with an isovalue of 0.04. Green and purple colors correspond to positive and negative phases of the orbital wavefunction, respectively. 24

Figure S25. Variation of Ru-Ru bond distances with the $N_{eq.}$-Ru-Ru-$N_{eq.}$ dihedral angle in compound [Ru_2Cl(D*p*-FPhF)$_4$] (**4**). 25

Table S8. Energies for the beta $\pi_{(2)}$-πL-Cl and $\pi_{(1)}$-πL orbitals, and the π-πL splitting, calculated for compounds **4** and **6** at two different torsion angles (8 and 15º, approximately). 25

S.8. M-M BOND DISTANCES AND TORSION ANGLES IN [M_2(DArF)$_4$] COMPOUNDS 26

Table S9. Average V-V distances and $\chi_{Neq.\text{-}V\text{-}V\text{-}Neq.}$ dihedral angles for [V_2(DArF)$_4$]. .. 26

Table S10. Average Cr-Cr distances and $\chi_{Neq.\text{-}Cr\text{-}Cr\text{-}Neq.}$ dihedral angles for [Cr_2(DArF)$_4$]. 26

Table S11. Average Mo-Mo distances and $\chi_{Neq.\text{-}Mo\text{-}Mo\text{-}Neq.}$ dihedral angles for [Mo_2(DArF)$_4$]. 26

Table S12. Average W-W distances and $\chi_{Neq.\text{-}W\text{-}W\text{-}Neq.}$ dihedral angles for [W_2(DArF)$_4$]. 27

Table S13. Average Rh-Rh distances and $\chi_{Neq.\text{-}Rh\text{-}Rh\text{-}Neq.}$ dihedral angles for [Rh_2(DArF)$_4$]. 27

S.9. M-M BOND DISTANCES AND TORSION ANGLES IN [M_2Cl(ap)$_4$] COMPOUNDS (M = Ru, Rh) 27

Table S14. Average Ru-Ru bond distances and $\chi_{Neq.\text{-}Ru\text{-}Ru\text{-}Neq.}$ angles for [Ru_2Cl(ap)$_4$]-type compounds (ap = anilinopyridinate). 27

Table S15. Average Rh-Rh bond distances and $\chi_{Neq.-Rh-Rh-Neq.}$ angles for $[Rh_2Cl(ap)_4]$-type compounds (ap = anilinopyridinate). 28

Figure S25. Variation of Rh-Rh (squares) and Ru-Ru (circles) bond distances with the $N_{eq.}$-M-M-$N_{eq.}$ dihedral angle in compounds with general formula $[M_2Cl(ap)_4]$ (ap = anilinopyridinate). The light dashed lines are guides to the eye. The distances and torsion values were obtained from the literature 28

S.10. REFERENCES 28

S.1. EXPERIMENTAL SECTION

General procedure. $[Ru_2Cl(O_2CCH_3)_4]$, *N,N'*-bis(3,5-dimethylphenyl)formamidine (HD*m*-XylF), *N,N'*-bis(3-methylphenyl)formamidine (HD*m*-TolF), *N,N'*-bis(4-methoxyphenyl)formamidine (HD*p*-AniF), *N,N'*-bis(4-fluorophenyl)formamidine (HD*p*-FPhF) and *N,N'*-bis(4-methylphenyl)formamidine (HD*p*-TolF) and were prepared following similar procedures to the ones described in the literature.[1], [2] *N,N'*-diphenylformamidine (HDPhF) was obtained from commercial sources and recrystalized in dichloromethane. The rest of reactants and solvents used in all the synthetic procedures described in this paper, were also obtained from commercial sources but used as received. Elemental analyses were carried out by the Microanalytical Service of the Complutense University of Madrid. Electrospray ionization (ESI^+) mass spectra were collected using an ion trap analyser HCT Ultra (Bruker Daltonics) mass spectrometer, in the Mass Spectrometry Service of the Complutense University of Madrid. The calculated C, H and N percentages and mass peaks were obtained using the MASAS software,[3] which considers the natural abundance of the different isotopes. Diffuse reflectance spectra were measured in double beam Cary 5G spectrophotometer. The powder sample was placed in a Praying Mantis Accessory, and Teflon was measured as a standard.

Synthesis of $[Ru_2Cl(Dm\text{-}XylF)_4]$ (1), **$[Ru_2Cl(Dm\text{-}TolF)_4]$ (2), $[Ru_2Cl(Dp\text{-}AniF)_4]$ (3), $[Ru_2Cl(Dp\text{-}FPhF)_4]$ (4)**, **$[Ru_2Cl(DPhF)_4]$ (5)** and **$[Ru_2Cl(Dp\text{-}TolF)_4]$ (6).** A mixture of 0.30 g (0.63 mmol) of $[Ru_2Cl(O_2CCH_3)_4]$, 0.15 g (3.54 mmol) of LiCl and 0.5 mL (3.60 mmol) of NEt_3 were suspended in 16 mL of THF with an excess of *N,N'*-diarylformamidine (at least four times the equivalent amount of $[Ru_2Cl(O_2CCH_3)_4]$). The reaction mixture was introduced inside an 85 mL Teflon vessel provided with a magnetic stir bar. This vessel was placed in the microwave oven, and the reaction mixture was heated for 4 hours at 130 ºC and cooled down in 1 hour to room temperature. The reaction

mixture was then filtered under vacuum, and the obtained dark solid in synthesis of **3** (blue), **4**, **5** and **6** (green) was washed with cold methanol until the filtering was colorless. In the case of synthesis of compound **1**, the filtered purple solution was dried under vacuum and then extracted, filtered, and washed with a large amount of acetone, until the filtering was colorless. Lastly, for compound **2**, the green filtered solution was dried under vacuum, giving rise to a purple solid which was then extracted filtered and washed with cold methanol (5 × 3 mL) and Et_2O (5 × 2 mL). All the synthesized compounds were dried under vacuum.

[Ru_2Cl(D*m*-XylF)$_4$] (1) Yield: 0.61 g (78%). Anal. Found (calculated) for $H_{76}C_{68}N_8ClRu_2$·acetone (1301.091 g/mol): C, 65.54 (65.71); H, 6.35 (6.16); N, 8.61 (9.01). MS (ESI^+) *m/z*: 1103 [**1**-Cl-C_8H_8+H]$^+$ (3%); 1208 [**1**-Cl]$^+$ (100%). Crystals suitable for X-ray diffraction were obtained by layering hexane over a solution of **1** in CH_2Cl_2.

[Ru_2Cl(D*m*-TolF)$_4$] (2) Yield: 0.29 g (41%). Anal. Found (calculated) for $H_{60}C_{60}N_8ClRu_2$·0.5Et_2O (1167.855 g/mol): C, 63.77 (63.73); H, 5.61 (5.35); N, 9.59 (9.91). MS (ESI^+) *m/z*: 1009 [**2**-Cl-C_7H_7+H]$^+$ (3%); 1096 [**2**-Cl]$^+$ (100%). Crystals suitable for X-ray diffraction were obtained by slow evaporation of a solution of **2** in hexane.

[Ru_2Cl(D*p*-AniF)$_4$] (3) Yield: 0.51 g (64%). Anal. Found (calculated) for $H_{60}C_{60}N_8O_8ClRu_2$ (1258.789 g/mol): C, 57.12 (57.25); H, 4.85 (4.80); N, 8.73 (8.90). MS (ESI^+) *m/z*: 1118 [**3**-Cl-C_7H_7O+H]$^+$ (7%); 1224 [**3**-Cl]$^+$ (100%). Crystals suitable for X-ray diffraction were obtained by layering hexane over a solution of **3** in CH_2Cl_2.

[Ru_2Cl(D*p*-FPhF)$_4$] (4) Yield: 0.33 g (45%). Anal. Found (calculated) for $H_{36}C_{52}N_8F_8ClRu_2$·MeOH (1194.542 g/mol): C, 53.39 (53.29); H, 3.36 (3.38); N, 9.30 (9.38). MS (ESI^+) *m/z*: 1035 [**4**-Cl-C_6H_4F+H]$^+$ (3%); 1128 [**4**-Cl]$^+$ (100%).

[Ru_2Cl(DPhF)$_4$] (5) Yield: 0.39 g (61%). Anal. Found (calculated) for $H_{44}C_{52}N_8ClRu_2$ (1018.577 g/mol): C, 61.26 (61.32); H, 4.75 (4.35); N, 10.24 (11.00). MS (ESI^+) *m/z*: 909 [**5**-Cl-C_6H_5+H]$^+$ (3%); 984 [**5**-Cl]$^+$ (100%).

[Ru_2Cl(D*p*-TolF)$_4$] (6) Yield: 0.42 g (59%). Anal. Found (calculated) for $H_{60}C_{60}N_8ClRu_2$·THF (1202.901 g/mol): C, 63.84 (63.90); H, 5.68 (5.70); N, 8.93 (9.32). MS (ESI$^+$) *m/z*: 1008 [**6**-Cl-C_7H_7+H]$^+$ (2%); 1096 [**6**-Cl]$^+$ (100%).

S.2. MASS SPECTROMETRY

This technique is especially useful for detecting impurities in this type of complexes. The simplicity of the spectra clearly indicates the existence of a single type of diruthenium species.

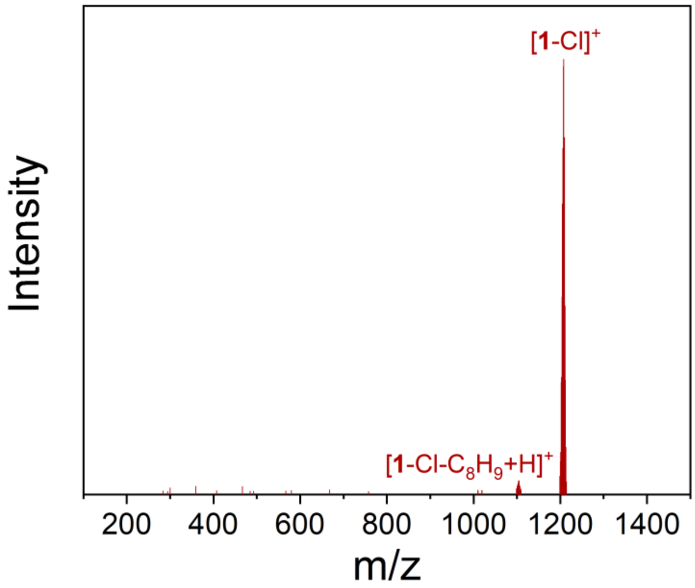

Figure S1. Positive ion ESI mass spectrum of [Ru_2Cl(D*m*-XylF)$_4$] (**1**).

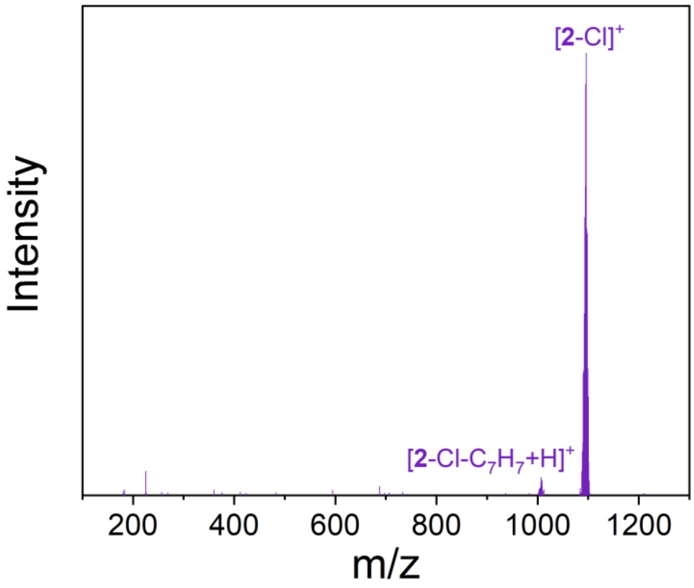

Figure S2. Positive ion ESI mass spectrum of [Ru_2Cl(D*m*-TolF)$_4$] (**2**).

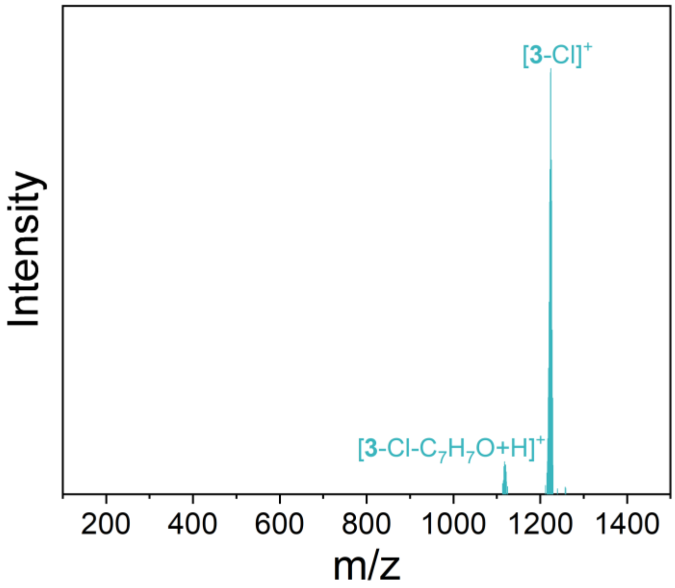

Figure S3. Positive ion ESI mass spectrum of [Ru_2Cl(D*p*-AniF)$_4$] (**3**).

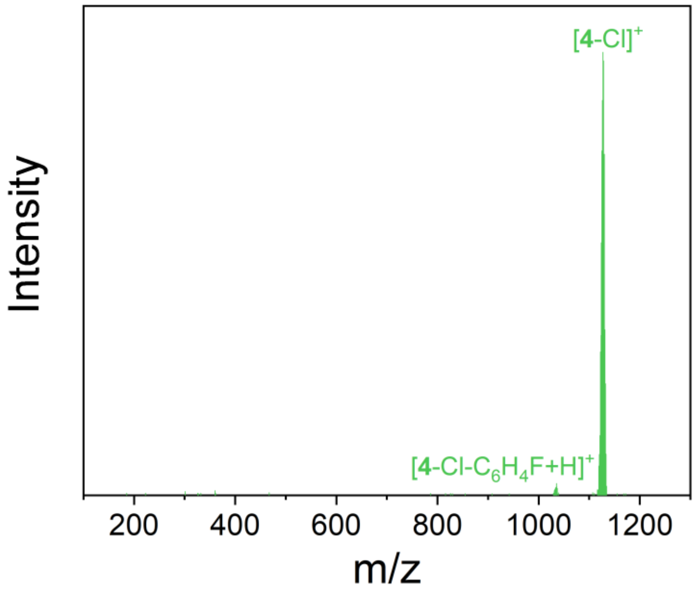

Figure S4. Positive ion ESI mass spectrum of $[Ru_2Cl(Dp\text{-}FPhF]_4$ (**4**).

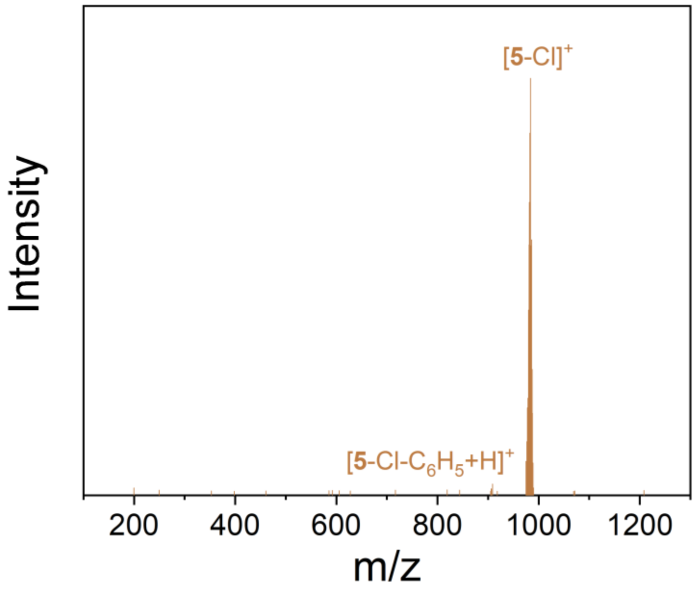

Figure S5. Positive ion ESI mass spectrum of $[Ru_2Cl(DPhF)_4]$ (**5**).

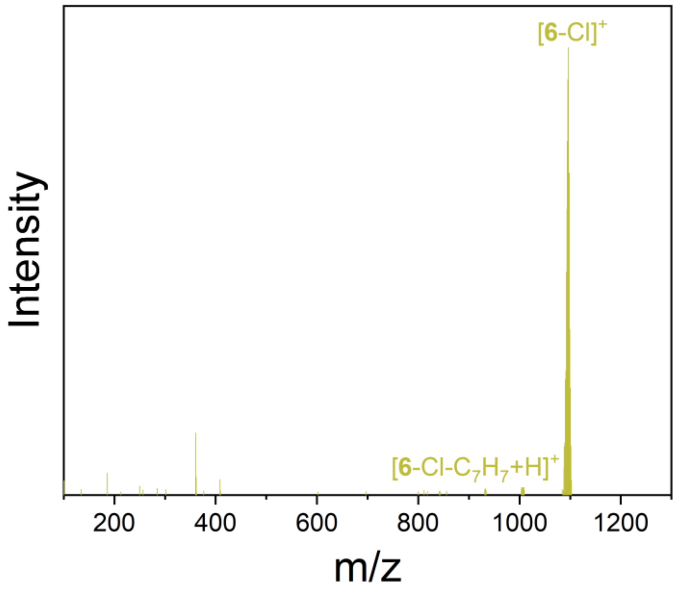

Figure S6. Positive ion ESI mass spectrum of $[Ru_2Cl(Dp\text{-}TolF)_4]$ (**6**).

S.3. SINGLE CRYSTAL X-RAY DIFFRACTION

Single crystals of compounds **1**, **2** and **3** were obtained as previously described in section S1. Crystal structures are represented using VESTA.[4]

$[Ru_2Cl(Dm\text{-}XylF)_4]\cdot 1.82H_2O$ ($1\cdot 1.82H_2O$) (CCDC 2301197). A dark purple, plate-like specimen of **$1\cdot 1.82H_2O$**, with approximate dimensions 0.029 mm × 0.079 mm × 0.081 mm, was used for the X-ray crystallographic analysis. The X-ray intensity data were measured in a Bruker Kappa Apex II diffractometer, equipped with a Mo sealed tube (λ = 0.71073 Å).

The integration of the data using a tetragonal unit cell yielded a total of 67305 reflections to a maximum θ angle of 25.37° (0.83 Å resolution), of which 3140 were independent (average redundancy 21.435, completeness = 99.8%, R_{int} = 4.96%, R_{sig} = 1.64%) and 2360 (75.16%) were greater than $2\sigma(F^2)$.

The final cell constants of a = 15.9025(3) Å, b = 15.9025(3) Å, c = 27.0253(8) Å, volume = 6834.4(3) Å^3, are based upon the refinement of the XYZ-centroids of 9980 reflections above 20 σ(I) with 5.923° < 2θ < 50.44°. Data were corrected for absorption effects using the Multi-Scan method (SADABS). The ratio of minimum to maximum apparent

transmission was 0.910. The calculated minimum and maximum transmission coefficients (based on crystal size) are 0.9590 and 0.9850.

The structure was solved and refined using the Bruker SHELXTL Software Package, using the space group *P4/ncc*, with Z = 4 for the formula unit, $C_{68}H_{76}ClN_8O_{1.82}Ru_2$. The final anisotropic full-matrix least-squares refinement on F^2 with 199 variables converged at R_1 = 4.53%, for the observed data and wR^2 = 20.93% for all data. The goodness-of-fit was 1.299. The largest peak in the final difference electron density synthesis was 1.327 $e^-/Å^3$ and the largest hole was -1.207 $e^-/Å^3$ with an RMS deviation of 0.301 $e^-/Å^3$. On the basis of the final model, the calculated density was 1.236 g/cm^3 and F(000), 2638 e^-.

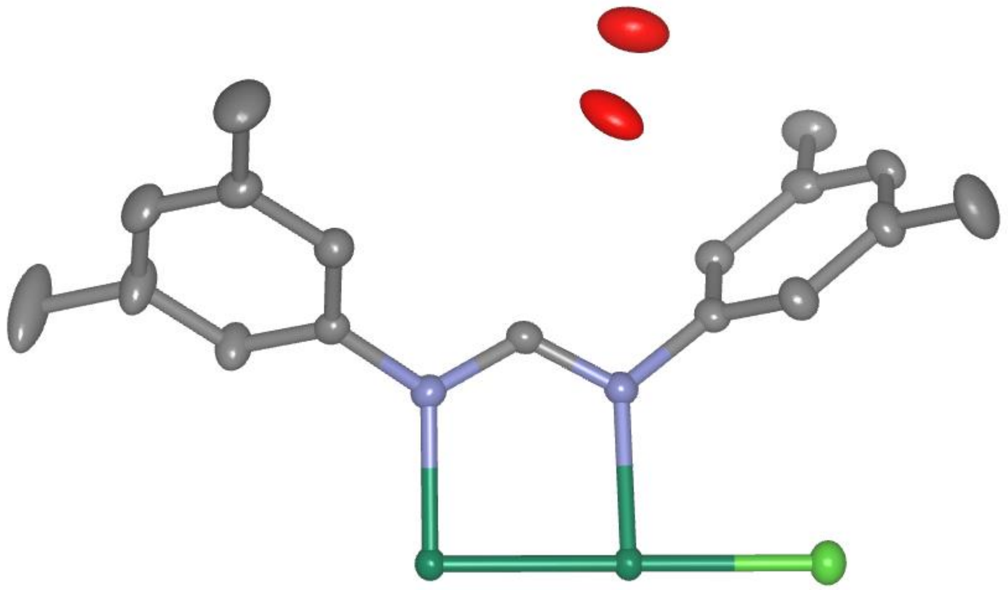

Figure S7. Asymmetric unit of **1·1.82H$_2$O**. Hydrogen atoms are omitted for clarity. Color code: dark green for Ru, light green for Cl, red for O, blue for N and grey for C. The ellipsoids are represented with a probability of 30%.

Table S1. Sample and crystal data for **1·1.82H$_2$O**.

**Empirical formula**	$C_{68}H_{76}ClN_8O_{1.82}Ru_2$
**Formula weight/g·mol^{-1}**	1272.07
**Temperature/K**	250(2)
**Crystal system**	tetragonal
**Space group**	*P*4/*ncc*
***a*/Å**	15.9025(3)
***b*/Å**	15.9025(3)
***c*/Å**	27.0253(8)
**α/°**	90
**β/°**	90
**γ/°**	90
**Volume/Å^3**	6834.4(3)
**Z**	4

**ρ_{calc}/g cm^{-3}**	1.236
**μ/mm^{1}**	0.527
**F(000)**	2638
**Crystal size/mm^3**	0.029 × 0.079 × 0.081
**Radiation**	CuKα (λ = 1.54178)
**Θ range for data collection/°**	1.51 to 25.37
**Index ranges**	-18 ≤h≤ 19, -19 ≤ k ≤ 19, -32 ≤ l ≤ 32
**Reflections collected**	67305
**Independent reflections**	3140 [R_{int} = 0.0496]
**Data/restraints/parameters**	3140 / 166 / 199
**Goodness-of-fit on F^2**	1.299
**Final R indexes [I>=2σ (I)]**	R_1 = 0.0453, wR_2 = 0.1658
**Final R indexes [all data]**	R_1 = 0.0781, wR_2 = 0.2093
**Largest diff. peak/hole / e Å^{-3}**	1.327 / -1.207

[Ru_2Cl(D*m*-TolF)$_4$]·0.5H_2O (2·0.5H_2O) (CCDC 2301198). A dark blue, plate-like specimen of **2·0.5H_2O**, with approximate dimensions 0.001 mm x 0.034 mm x 0.050 mm, was used for the X-ray crystallographic analysis. The X-ray intensity data were measured in a Rigaku XtaLAB Synergy-DW, equipped with a Cu rotating anode (λ = 1.54178 Å).

The integration of the data using a triclinic unit cell yielded a total of 21183 reflections to a maximum θ angle of 66.63° (0.84 Å resolution), of which 10132 were independent (average redundancy 2.091, completeness = 98.5%, R_{int} = 5.15%, R_{sig} = 7.68%) and 6972 (68.81%) were greater than 2σ(F^2).

The final cell constants of a = 13.3765(5) Å, b = 13.4182(4) Å, c = 17.2835(8) Å, α = 106.860(3)°, β = 101.153(4)°, γ = 90.519(3)°, volume = 2905.8(2) Å^3, are based upon the refinement of the XYZ-centroids of 2059 reflections above 20 σ(I) with 5.147° < 2θ < 36.90°. Data were corrected for absorption effects using analytical methods. The ratio of minimum to maximum apparent transmission was 0.799. The calculated minimum and maximum transmission coefficients (based on crystal size) are 0.72 and 0.97.

The structure was solved and refined using the Bruker SHELXTL Software Package, using the space group *P*-1, with Z = 2 for the formula unit, $C_{60}H_{60}ClN_8O_{0.50}Ru_2$. The final anisotropic full-matrix least-squares refinement on F^2 with 656 variables converged at R_1 = 6.81%, for the observed data and wR_2 = 20.95% for all data. The goodness-of-fit was 1.035. The largest peak in the final difference electron density synthesis was 4.736 e$^-$/Å^3 and the largest hole was -1.370 e$^-$/Å^3 with an RMS deviation of 0.180 e$^-$/Å^3. On the basis of the final model, the calculated density was 1.302 g/cm^3 and F(000), 1170 e$^-$.

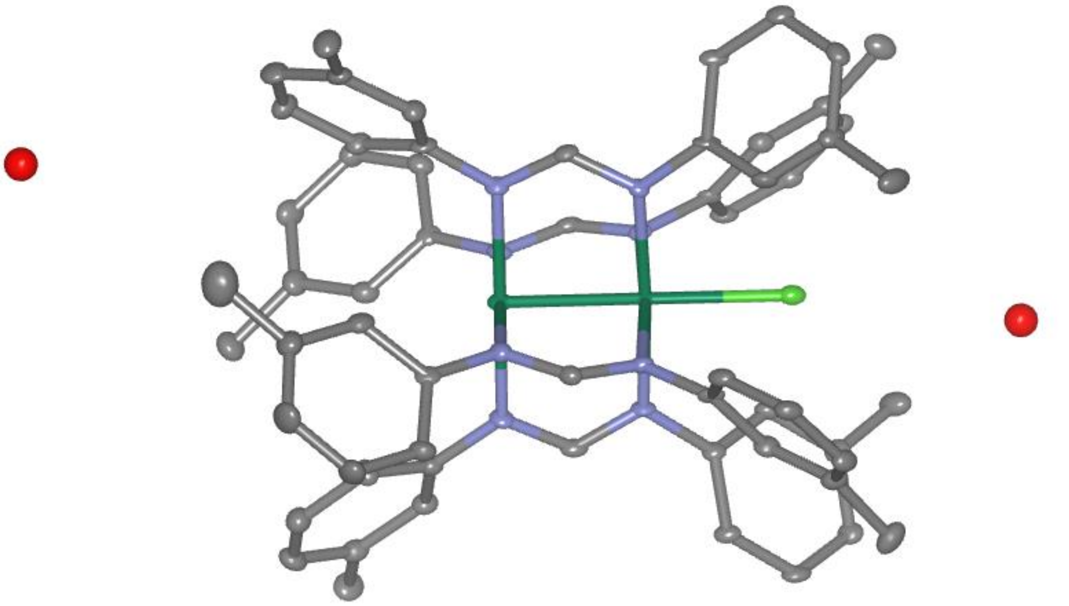

Figure S8. Asymmetric unit of **2·0.5 H_2O**. Hydrogen atoms are omitted for clarity. Color code: dark green for Ru, light green for Cl, red for O, blue for N and grey for C. The ellipsoids are represented with a probability of 30%.

Table S2. Sample and crystal data for **2·0.5H_2O**.

**Empirical formula**	$C_{60}H_{60}ClN_8O_{0.50}Ru_2$
**Formula weight/g·mol^{-1}**	1138.75
**Temperature/K**	100(2)
**Crystal system**	triclinic
**Spacegroup**	*P*-1
***a*/Å**	13.3765(5)
***b*/Å**	13.4182(4)
***c*/Å**	17.2835(8)
**α/°**	106.860(3)
**β/°**	101.153(4)
**γ/°**	90.519(3)
**Volume/Å^3**	2905.8(2)
**Z**	2
**ρ_{calc}/g cm^{-3}**	1.302
**μ/mm^1**	4.970
**F(000)**	1170
**Crystal size/mm^3**	0.001 × 0.034 × 0.050
**Radiation**	CuK_α (λ = 1.54178)
**Θ range for data collection/°**	2.73 to 66.63
**Index ranges**	-15 ≤ h ≤ 15, -8 ≤ k ≤ 8, -20≤ l ≤19
**Reflections collected**	21183
**Independent reflections**	10132 [R_{int} = 0.0515]
**Data/restraints/parameters**	10132 / 0 / 656
**Goodness-of-fit on F^2**	1.035
**Final R indexes [I>=2σ (I)]**	R_1 = 0.0681, wR_2 = 0.1876
**Final R indexes [all data]**	R_1 = 0.1017, wR_2 = 0.2095
**Largest diff. peak/hole / e Å^{-3}**	4.736/-1.370

[Ru_2Cl(D*p*-AniF)$_4$]·0.5CH_2Cl_2 (3·0.5CH_2Cl_2) (CCDC 2301199). A dark purple, plate-like specimen of **3·0.5CH_2Cl_2**, with approximate dimensions 0.030 mm x 0.140 mm x 0.140 mm, was used for the X-ray crystallographic analysis. Bruker D8 Venture diffractometer, equipped with a Cu microfocus sealed tube (λ = 1.54178 Å).

The integration of the data using a tetragonal unit cell yielded a total of 36715 reflections to a maximum θ angle of 68.25° (0.83 Å resolution), of which 2682 were independent (average redundancy 13.689, completeness = 99.8%, R_{int} = 3.39%, R_{sig} = 1.41%) and 2549 (95.04%) were greater than 2σ(F^2).

The final cell constants of *a* = 13.2403(2) Å, *b* = 13.2403(2) Å, *c* = 16.6670(3) Å, volume = 2921.82(10) Å^3, are based upon the refinement of the XYZ-centroids of reflections above 20 σ(I). Data were corrected for absorption effects using the Multi-Scan method (SADABS). The ratio of minimum to maximum apparent transmission was 0.700. The calculated minimum and maximum transmission coefficients (based on crystal size) are 0.6000 and 0.8500.

The structure was solved and refined using the Bruker SHELXTL Software Package, using the space group *P4/n*, with Z = 2 for the formula unit, $C_{60.50}H_{60}Cl_2N_8O_8Ru_2$. The final anisotropic full-matrix least-squares refinement on F^2 with 201 variables converged at R_1 = 2.23%, for the observed data and wR_2 = 6.77% for all data. The goodness-of-fit was 1.016. The largest peak in the final difference electron density synthesis was 0.559 e$^-$/Å^3 and the largest hole was -0.381 e$^-$/Å^3 with an RMS deviation of 0.062 e$^-$/Å^3. On the basis of the final model, the calculated density was 1.478 g/cm^3 and F(000), 1330 e$^-$.

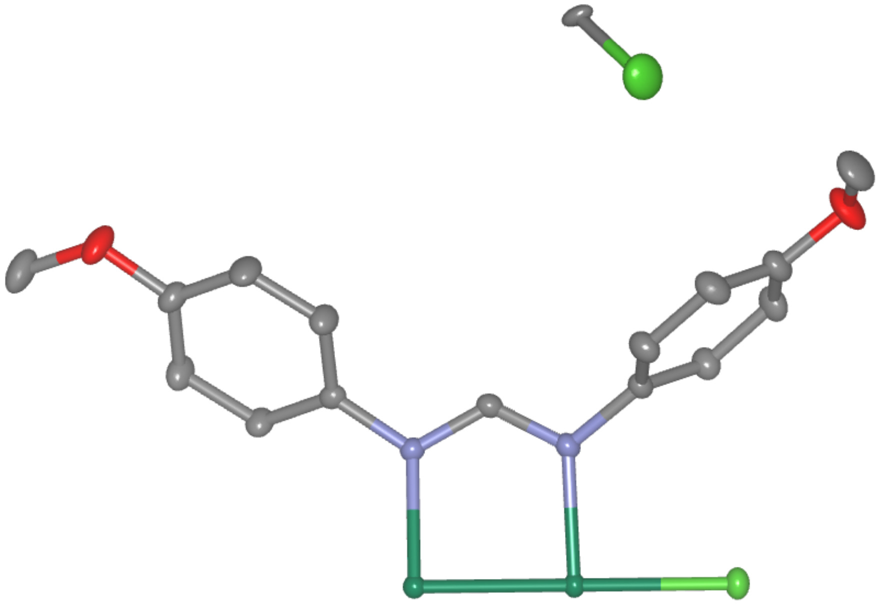

Figure S9. Asymmetric unit of **3·0.5CH$_2$Cl$_2$**. Hydrogen atoms are omitted for clarity. Color code: dark green for Ru, light green for Cl, red for O, blue for N and grey for C. The ellipsoids are represented with a probability of 30%.

Table S3. Sample and crystal data for **3·0.5CH$_2$Cl$_2$**.

**Empirical formula**	$C_{60.50}H_{60}Cl_2N_8O_8Ru_2$
**Formula weight/g·mol^{-1}**	1300.20
**Temperature/K**	297(2)
**Crystal system**	tetragonal
**Space group**	*P*4/*n*
***a*/Å**	13.2403(2)
***b*/Å**	13.2403(2)
***c*/Å**	16.6670(3)
**α/°**	90
**β/°**	90
**γ/°**	90
**Volume/Å^3**	2921.8(1)
**Z**	2
**ρ_{calc}/g cm^{-3}**	1.478
**μ/mm^1**	5.529
**F(000)**	1330
**Crystal size/mm^3**	0.030 × 0.140 × 0.140
**Radiation**	MoKα (λ = 0.71073)
**Θ range for data collection/°**	2.65 to 68.25
**Index ranges**	-15 ≤ h ≤ 14, -15 ≤ k ≤ 15, -20 ≤ l ≤ 20
**Reflections collected**	36715
**Independent reflections**	2682 [R_{int} = 0.0339]
**Data/restraints/parameters**	2682 / 1 / 201
**Goodness-of-fit on F^2**	1.016
**Final R indexes [I>=2σ (I)]**	R_1 = 0.0223, wR_2 = 0.0659
**Final R indexes [all data]**	R_1 = 0.0240, wR_2 = 0.0677
**Largest diff. peak/hole / e Å^{-3}**	0.559 / -0.381

S.4. M-M BOND DISTANCES AND TORSION ANGLES IN $[Ru_2Cl(DArF)_4]$ COMPOUNDS

Table S4. Ru-Ru bond distances and $N_{eq.}$-Ru-Ru-$N_{eq.}$ dihedral angles for compounds with general formula $[Ru_2Cl(DArF)_4]$.

	**dRu-Ru / Å**	$\chi_{Neq.-Ru-Ru-Neq.}$ **/ deg**
$[Ru_2Cl(D$*m*-XylF$)_4]$ (**1**)	2.3785(9)	3.2(5)
$[Ru_2Cl(D$*m*-TolF$)_4]$ (**2**)	2.3744(8)	7.9(2)
	2.3900(3)	7.8(3)
$[Ru_2Cl(D$*p*-AniF$)_4]$ [5], [6] (**3**)	2.3960(5)	7.36(6)
	2.328(1)	16.4(4)
$[Ru_2Cl(D$*p*-FPhF$)_4]$ [7] (**4**)	2.3530(9)	12.4(2)
$[Ru_2Cl(DPhF)_4]$ [6], [8] (**5**)	2.344(1)	13.0(2)
	2.339(4)	14.5(2)
$[Ru_2Cl(D$*p*-TolF$)_4]$ [9] (**6**)	2.370(2)	15.2(3)
$[Ru_2Cl(D$*m*-AniF$)_4]$ [10]	2.3398(4)	10.1(1)
$[Ru_2Cl(D$*m*,*m*-$Cl_2PhF)_4]$ [11]	2.360(2)	1.8(2)
$[Ru_2Cl(TPG)_4]$ [12]	2.3039(8)	18.6(7)
$[Ru_2Cl(D$*m*,*m*-$Cl_2PhF)_3(D$*p*-I-PhF$)]$ [13]	2.3374(7)	13.5(2)
$[Ru_2Cl(D$*m*-AniF$)_3(D$*p*-I-PhF$)]$ [13]	2.3302(6)	11.8(3)
$[Ru_2Cl(D$*m*,*m*-$Cl_2PhF)_3(D$*p*-(Ph)-PhF$)]$ [13]	2.3332(9)	10.4(3)

Table S5. NCN bite angles, distances between nitrogen binding atoms from the equatorial ligands, and N-Ru-Ru angles for compounds with general formula $[Ru_2Cl(DArF)_4]$.

	γ_{NCN} **/ deg**	**dN···N / Å**	α_{NRuRu} **/ deg**	$\alpha_{NRuRu(-Cl)}$ **/ deg**
$[Ru_2Cl(D$*m*-XylF$)_4]$ (**1**)	122.8(4)	2.318(4)	90.41(9)	87.88(9)
$[Ru_2Cl(D$*m*-TolF$)_4]$ (**2**)	123.32(7)	2.325(9)	90.15(18)	88.01(18)
	123.48(19)	2.326(2)	89.94(4)	87.82(4)
$[Ru_2Cl(D$*p*-AniF$)_4]$ [5], [6] (**3**)	123.2(2)	2.322(2)	89.72(4)	87.80(4)
	121.9(1)	2.32(1)	90.0(3)	87.6(2)
$[Ru_2Cl(D$*p*-FPhF$)_4]$ [7] (**4**)	122.8(7)	2.309(6)	90.4(1)	87.5(1)
$[Ru_2Cl(DPhF)_4]$ [6], [8] (**5**)	122.4(6)	2.314(7)	90.5(1)	87.4(1)
	121.9(5)	2.317(7)	90.4(1)	87.3(1)
$[Ru_2Cl(D$*p*-TolF$)_4]$ [9] (**6**)	122.3(8)	2.314(7)	90.5(1)	87.7(2)
$[Ru_2Cl(D$*m*-AniF$)_4]$ [10]	122.7(4)	2.309(4)	90.70(8)	87.63(8)
$[Ru_2Cl(D$*m*,*m*-$Cl_2PhF)_4]$ [11]	122.4(6)	2.308(8)	90.43(1)	88.13(1)
$[Ru_2Cl(TPG)_4]$ [12]	119.0(2)	2.309(3)	90.21(5)	87.18(5)
$[Ru_2Cl(D$*m*,*m*-$Cl_2PhF)_3(D$*p*-I-PhF$)]$ [13]	121.9(4)	2.315(6)	89.9(1)	88.0(1)
$[Ru_2Cl(D$*m*-AniF$)_3(D$*p*-I-PhF$)]$ [13]	123.3(8)	2.310(6)	90.6(1)	87.7(1)
$[Ru_2Cl(D$*m*,*m*-$Cl_2PhF)_3(D$*p*-(Ph)-PhF$)]$ [13]	122.1(9)	2.32(1)	90.6(3)	88.1(2)

S.5. MAGNETIC MEASUREMENTS

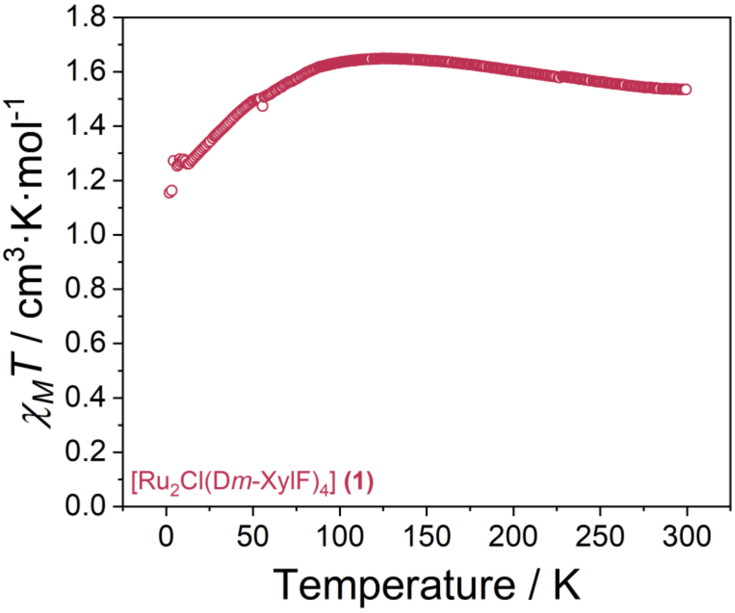

Figure S10. Temperature dependence of $\chi_M T$ for powder sample of [Ru_2Cl(D*m*-XylF)$_4$] (**1**).

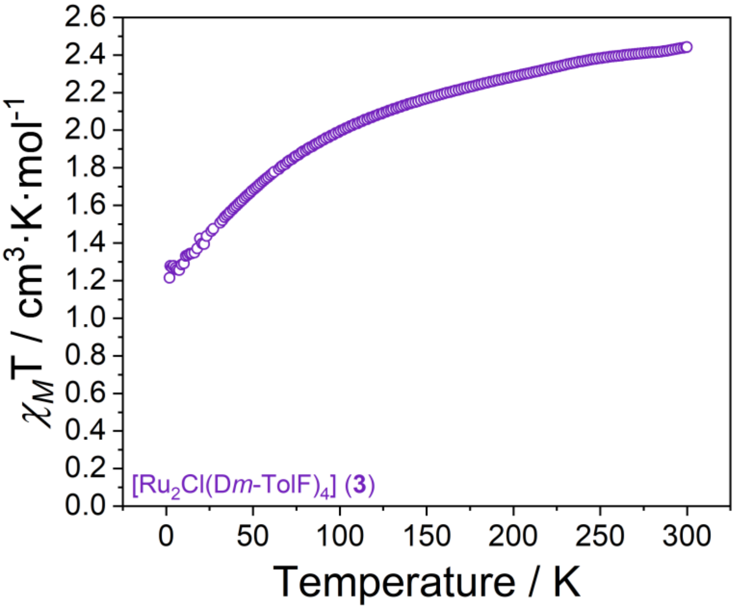

Figure S11. Temperature dependence of $\chi_M T$ for powder sample of [Ru_2Cl(D*m*-TolF)$_4$] (**2**).

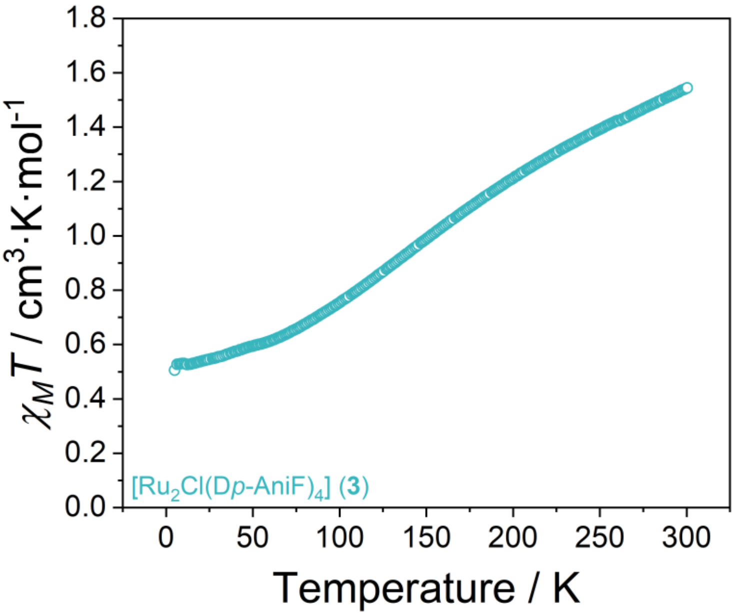

Figure S12. Temperature dependence of $\chi_M T$ for powder sample of $[Ru_2Cl(Dp\text{-AniF})_4]$ (**3**).

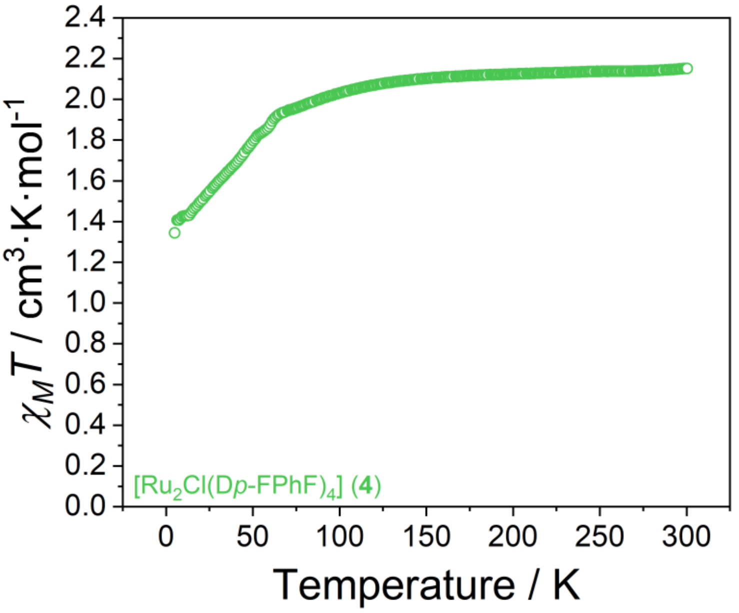

Figure S13. Temperature dependence of $\chi_M T$ for powder sample of $[Ru_2Cl(Dp\text{-FPhF})_4]$ (**4**).

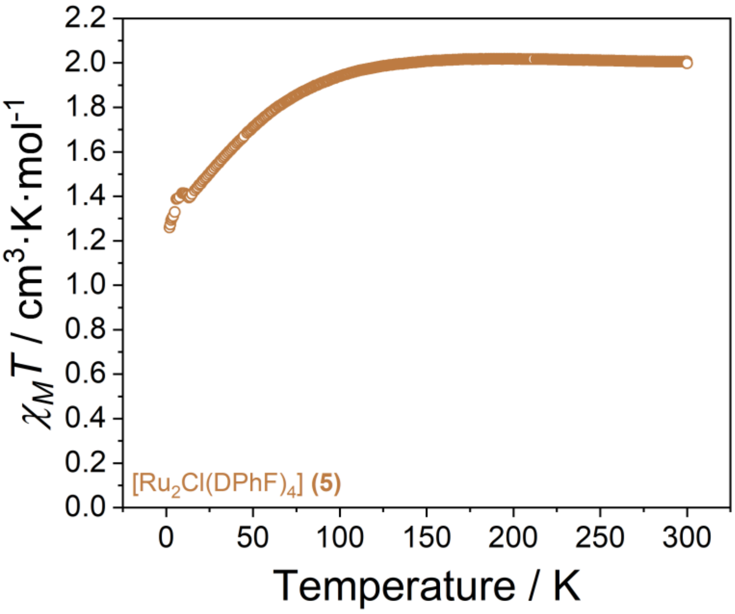

Figure S14. Temperature dependence of $\chi_M T$ for powder sample of $[Ru_2Cl(DPhF)_4]$ (**5**).

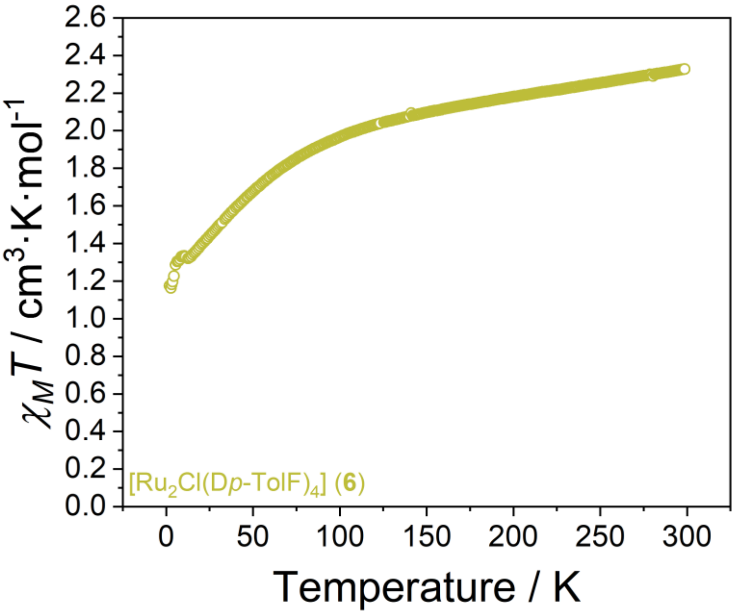

Figure S15. Temperature dependence of $\chi_M T$ for powder sample of $[Ru_2Cl(Dp\text{-}TolF)_4]$ (**6**).

S.6. DIFFUSE REFLECTANCE SPECTROSCOPY

The analysis of the diffuse reflectance spectra of compounds **1** – **6** was performed following three main steps. First, the base line was corrected, applying the same criterion for all cases. It was constructed based on a method derived from Rayleigh and Mie light scattering theory introduced by Leach *et al.*,[14] ensuring that the shape of the bands is as less modified as possible. The resulting baseline is subtracted from the spectra, which are then essentially rid any interference of the light scattering.[15] Then, the number of

contributions was decided by means of the second derivative method. Lastly, the optical absorption bands in each spectrum, which is represented in a wavenumber (cm^{-1}) scale instead of wavelength, are fitted to a Gaussian function.

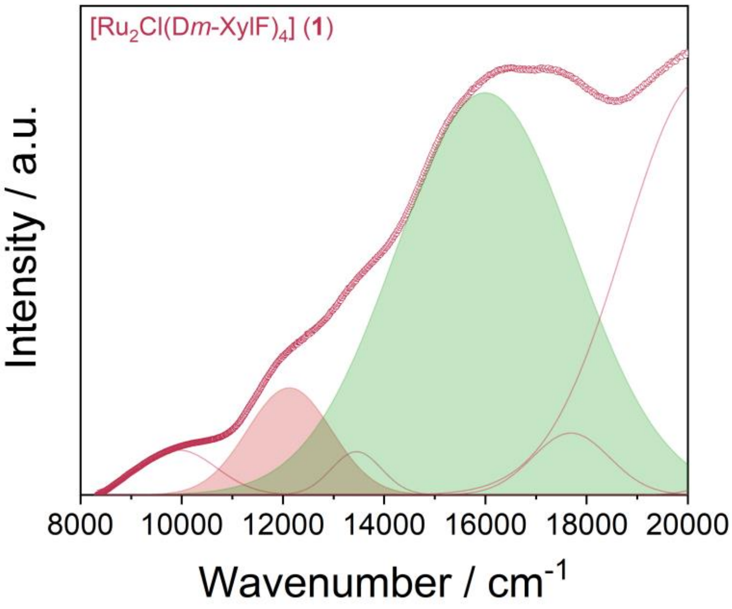

Figure S16. The Gaussian fit of the diffuse reflectance spectrum of compound [Ru_2Cl(D*m*-XylF)$_4$] (**1**). The δ(Ru_2) → π*(Ru_2) and π(Ru-N,Ru_2) → δ*(Ru_2) electronic transitions are represented in red and green, respectively.

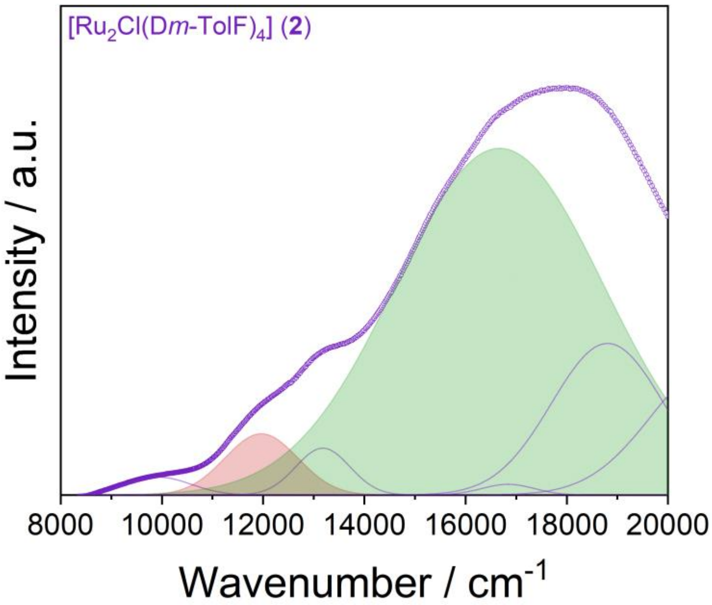

Figure S17. The Gaussian fit of the diffuse reflectance spectrum of compound [Ru_2Cl(D*m*-TolF)$_4$] (**2**). The δ(Ru_2) → π*(Ru_2) and π(Ru-N,Ru_2) → δ*(Ru_2) electronic transitions are represented in red and green, respectively.

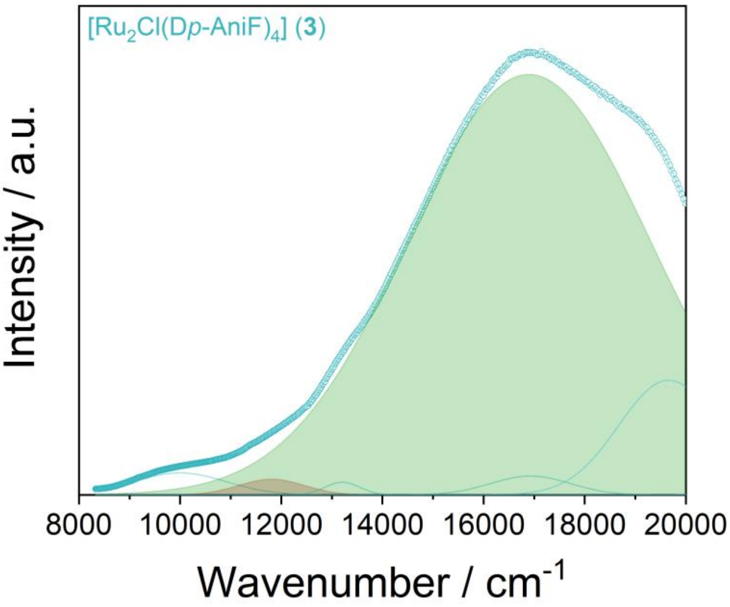

Figure S18. The Gaussian fit of the diffuse reflectance spectrum of compound [Ru_2Cl(D*p*-AniF)$_4$] (**3**). The δ(Ru_2) → π*(Ru_2) and π(Ru-N,Ru_2) → δ*(Ru_2) electronic transitions are represented in red and green, respectively.

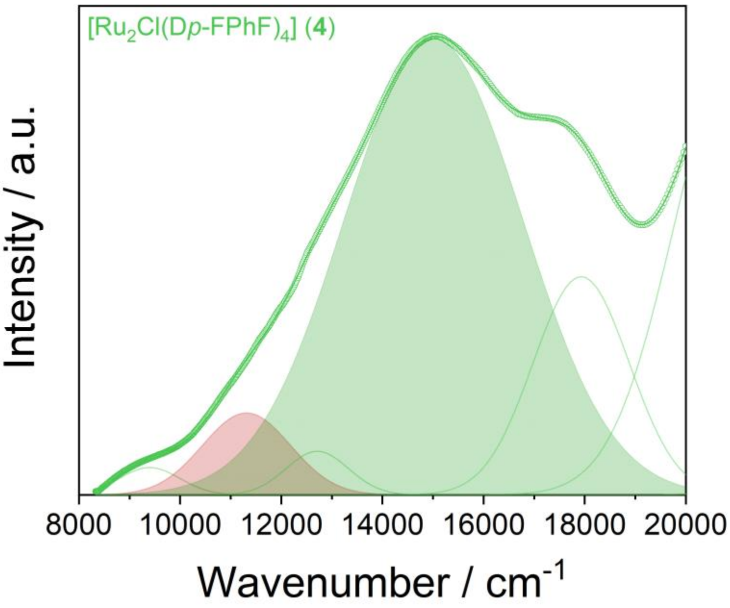

Figure S19. The Gaussian fit of the diffuse reflectance spectrum of compound [Ru_2Cl(D*p*-FPhF)$_4$] (**4**). The δ(Ru_2) → π*(Ru_2) and π(Ru-N,Ru_2) → δ*(Ru_2) electronic transitions are represented in red and green, respectively.

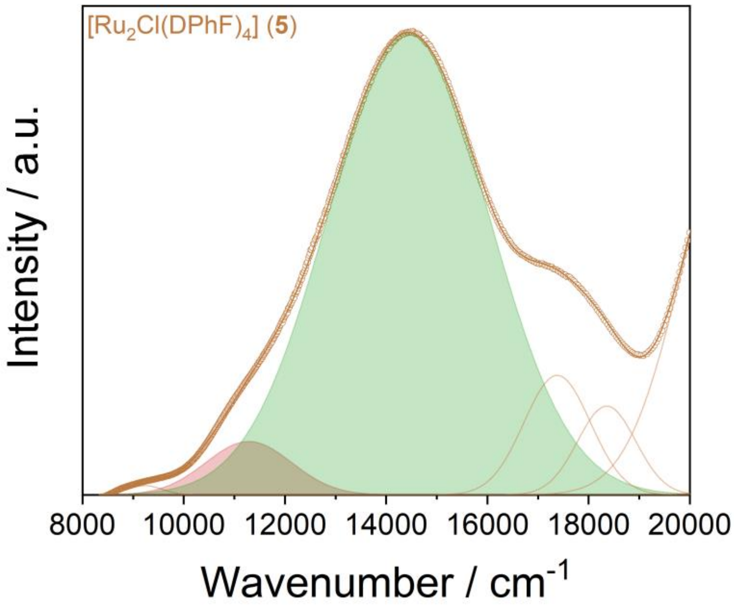

Figure S20. The Gaussian fit of the diffuse reflectance spectrum of compound $[Ru_2Cl(DPhF)_4]$ (**5**). The $\delta(Ru_2) \rightarrow \pi^*(Ru_2)$ and $\pi(Ru\text{-}N,Ru_2) \rightarrow \delta^*(Ru_2)$ electronic transitions are represented in red and green, respectively.

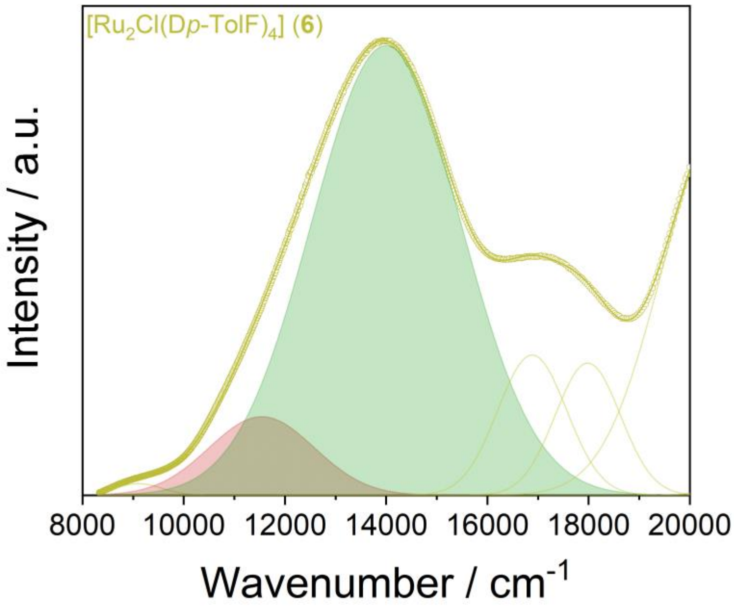

Figure S21. The Gaussian fit of the diffuse reflectance spectrum of compound $[Ru_2Cl(D\textit{p}\text{-}TolF)_4]$ (**6**). The $\delta(Ru_2) \rightarrow \pi^*(Ru_2)$ and $\pi(Ru\text{-}N,Ru_2) \rightarrow \delta^*(Ru_2)$ electronic transitions are represented in red and green, respectively.

Table S6. Tentative assignment for the observed bands in diffuse reflectance spectra of compounds **1** – **6**, based on previous calculations performed on $[Ru_2Cl(ap)_4]$ (ap = anilinopyridinate).[16]

	**Wavenumber / cm^{-1}**					
	**1**	**2**	**3**	**4**	**5**	**6**
$\delta(Ru_2) \rightarrow \pi^*(Ru_2)$	12,128	11,966	11,811	11,313	11,285	11,542

π(N) → π*(Ru_2)	13,454	13,182	13,208	12,714	-	-
π(Ru-N,Ru_2) → δ*(Ru_2)	15,994	16,675	16,898	15,011	14,441	14,002
σ(Ru_2/axial) → π*(Ru_2)	17,691	16,841	16,929	17,931	17,381	16,887
π*(Ru_2) → σ*(Ru_2/axial)	20,302	18,808	19,660	-	18,359	17,985
π(Ru_2) → π*(N)/δ*(Ru_2)	21,875	20,928	21,538	20,997	20,909	20,624
π(N/aryl) → σ*(Ru_2/axial)	-	-	-	22,704	22,606	22,401

S.7. DFT CALCULATIONS

All DFT calculations were performed with the Amsterdam Density Functional (ADF)[17] and QUILD[18] programs. Molecular orbitals were expanded in an uncontracted set of Slater type orbitals (STOs) of triple-ζ quality with double polarization functions (TZ2P).[19], [20] Core electrons were not treated explicitly during the geometry optimizations (frozen core approximation).[17] An auxiliary set of s, p, d, f, and g STOs was used to fit the molecular density and to represent the Coulomb and exchange potentials accurately for each SCF cycle.

The initial structure was optimized without any geometric restriction starting from the experimental X-ray molecular structure. Using this optimized structure as a model, we fixed the $N_{eq.}$-Ru-Ru-$N_{eq.}$ torsion angle at different values (from 0 to 25 degrees) and optimized the rest of the geometric degrees of freedom including the Ru-Ru distance. Geometries of the high spin state at various torsion angles were optimized with the QUILD[18] program using adapted delocalized coordinates until the maximum gradient component was less than 10^{-4} a.u. Energies and gradients were calculated using S12g,[21] in all cases by including solvation effects through the COSMO[22] dielectric continuum model with appropriate parameters for dichloromethane as solvent. For computing Gibbs free energies, all small frequencies were raised to 100 cm^{-1} in order to compensate for the breakdown of the harmonic oscillator model.[23], [24] Scalar relativistic corrections have been included self-consistently in all calculations by using the zeroth-order regular approximation (ZORA).[25] S12g calculations were performed with a Becke grid of VeryGood quality. All DFT calculations were performed using the unrestricted Kohn-Sham scheme.

All computational data have been uploaded (DOI: 10.19061/iochem-bd-4-68) onto the IOCHEM-BD platform (www.iochem-bd.org) to facilitate data exchange and dissemination, according to the FAIR principles[26] of OpenData sharing.

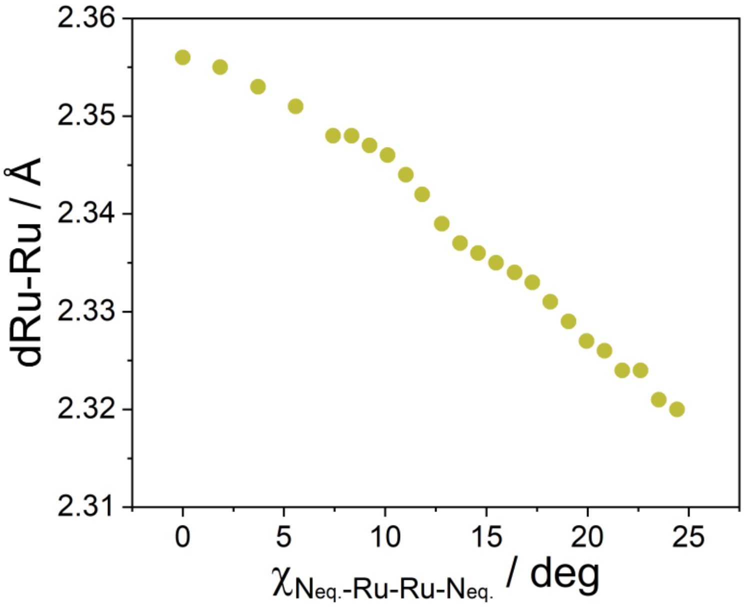

Figure S22. Variation of Ru-Ru bond distances with the $N_{eq.}$-Ru-Ru-$N_{eq.}$ dihedral angle in compound [Ru_2Cl(D*p*-TolF)$_4$] (**6**).

Table S7. Ru-Ru bond distances and $N_{eq.}$-Ru-Ru-$N_{eq.}$ dihedral angles angle in compound [Ru_2Cl(D*p*-TolF)$_4$] (**6**).

dRu-Ru / Å	$\chi_{Neq.-Ru-Ru-Neq.}$ / deg	dRu-Ru / Å	$\chi_{Neq.-Ru-Ru-Neq.}$ / deg
2.356	0.01	2.336	14.59
2.355	1.86	2.335	15.49
2.353	3.73	2.334	16.40
2.351	5.59	2.333	17.28
2.348	7.43	2.331	18.17
2.348	8.34	2.329	19.07
2.347	9.24	2.327	19.95
2.346	10.13	2.326	20.84
2.344	11.02	2.324	21.71
2.342	11.83	2.324	22.61
2.339	12.81	2.321	23.52
2.337	13.70	2.320	24.42

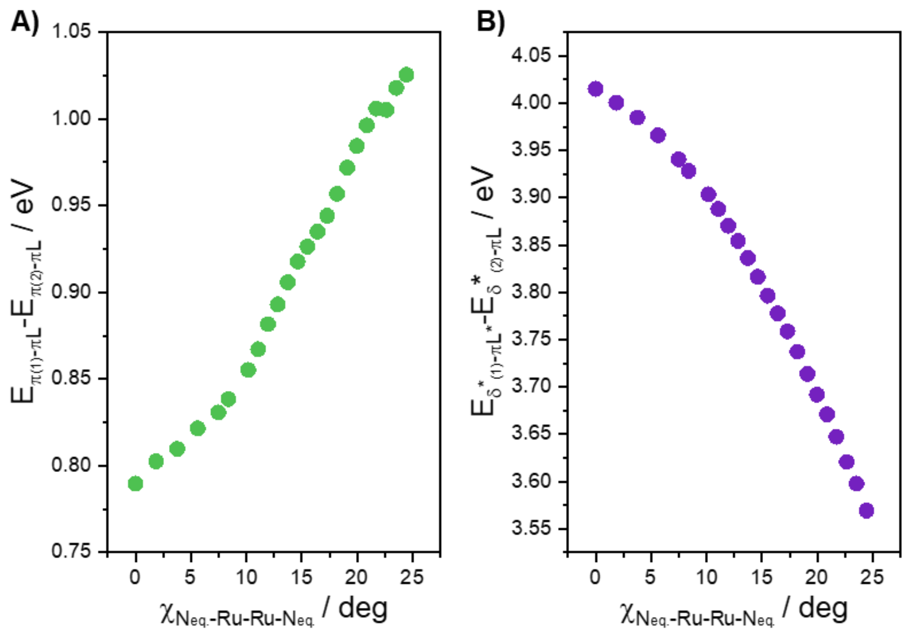

Figure S23. Splitting of the A) π-πL and B) δ*-πL molecular orbitals at various angles calculated for [Ru_2Cl(D*p*-TolF)$_4$] (**6**).

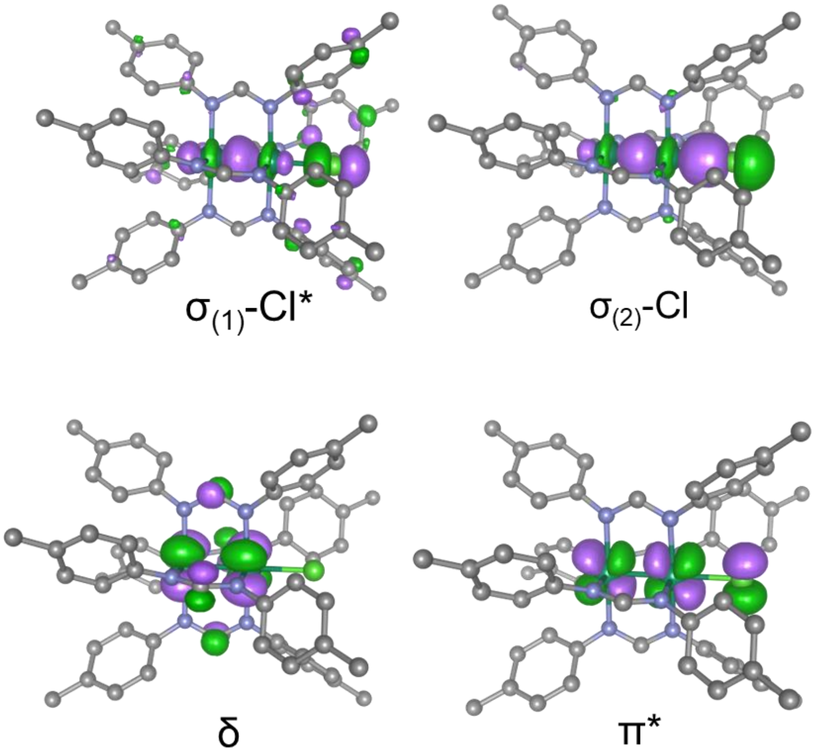

Figure S24. Isosurface maps of the Ru-Ru based molecular orbitals of compound [Ru_2Cl(D*p*-TolF)$_4$] (**6**), with an isovalue of 0.04. Green and purple colors correspond to positive and negative phases of the orbital wavefunction, respectively.

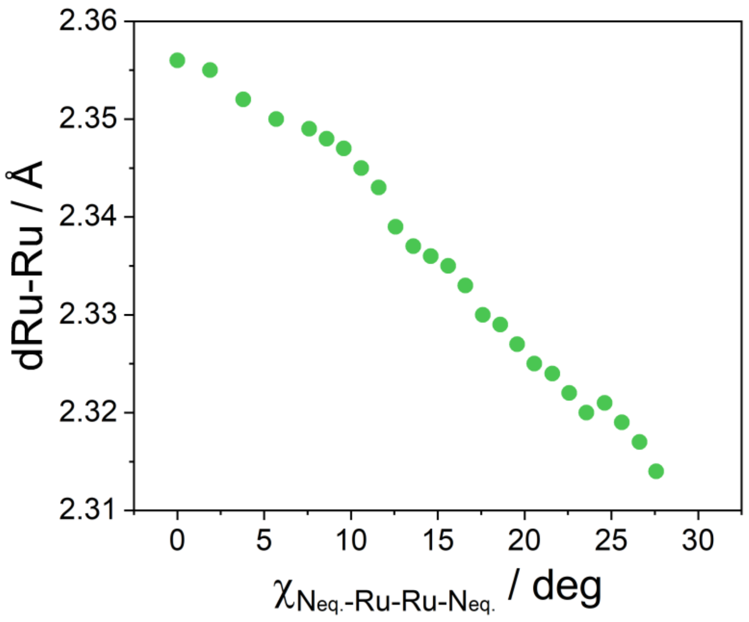

Figure S25. Variation of Ru-Ru bond distances with the $N_{eq.}$-Ru-Ru-$N_{eq.}$ dihedral angle in compound [$Ru_2Cl(Dp$-FPhF$)_4$] (**4**).

Table S8. Energies for the Ru_2 molecular orbitals, and the π-πL and δ*-πL splitting's, in eV, calculated for compounds **4** and **6** at two different torsion angles (8 and 15º, approximately).

	[$Ru_2Cl(Dp$-FPhF$)_4$] (**4**)	[$Ru_2Cl(Dp$-TolF$)_4$] (**6**)
	$\chi_{Neq.-Ru-Ru-Neq.} \approx 8^o$	
$\sigma_{(2)}$-Cl	-8.805	-8.550
$\delta^*_{(2)}$-πL	-7.847	-7.553
$\pi_{(2)}$-πL-Cl	-6.327	-6.077
$\sigma_{(1)}$-Cl*	-6.517	-6.097
$\pi_{(1)}$-πL	-5.495	-5.259
δ	-5.512	-5.282
π*-Cl*	-5.300	-5.067
$\delta^*_{(1)}$-πL*	-4.612	-4.379
π-πL splitting	0.832	0.818
δ*-πL splitting	3.235	3.174
	$\chi_{Neq.-Ru-Ru-Neq.} \approx 15^o$	
$\sigma_{(2)}$-Cl	-8.782	-8.551
$\delta^*_{(2)}$-πL	-7.743	-7.500
$\pi_{(2)}$-πL-Cl	-6.303	-6.090
$\sigma_{(1)}$-Cl*	-6.463	-6.111
$\pi_{(1)}$-πL	-5.420	-5.213
δ	-6.328	-5.254
π*-Cl*	-5.271	-5.074
$\delta^*_{(1)}$-πL*	-4.621	-4.418
π-πL splitting	0.882	0.877
δ*-πL splitting	3.122	3.082

S.8. M-M BOND DISTANCES AND TORSION ANGLES IN $[M_2(DArF)_4]$ COMPOUNDS

Table S9. Average V-V distances and $\chi_{Neq.\text{-}V\text{-}V\text{-}Neq.}$ dihedral angles for $[V_2(DArF)_4]$.

	**dV-V / Å**	$\chi_{Neq.\text{-}V\text{-}V\text{-}Neq.}$ **/ deg**
$[V_2$(D*p*-TolF)$_4]$ [27]	1.978	5.33
$[V_2$(DPhF)$_4]$ [28]	1.979	2.38
$[V_2$(D*p*-AniF)$_4]$ [28]	1.988	2.02
$[V_2$(D*p*-ClPhF)$_4]$ [28]	1.974	7.74
$[V_2$(D*p*-ClPhF)$_4]$ [28]	1.982	1.12
$[V_2$(TPG)$_4]$ [28]	1.952	13.96

Table S10. Average Cr-Cr distances and $\chi_{Neq.\text{-}Cr\text{-}Cr\text{-}Neq.}$ dihedral angles for $[Cr_2(DArF)_4]$.

	**dCr-Cr / Å**	$\chi_{Neq.\text{-}Cr\text{-}Cr\text{-}Neq.}$ **/ deg**
$[Cr_2$(D*p*-TolF)$_4]$ [29]	1.930	8.54
$[Cr_2$(D*o*-FPhF)$_4]$ [30]	1.968	3.83
$[Cr_2$(D*m*-FPhF)$_4]$ [30]	1.918	0.36
$[Cr_2$(D*p*-FPhF)$_4]$ [30]	1.916	5.23
$[Cr_2$(D*m*,*m*-F_2PhF)$_4]$ [30]	1.906	9.10
$[Cr_2$(DF_5PhF)$_4]$ [30]	2.012	13.22
$[Cr_2$(D*p*-(Ph)PhF)$_4]$ [30]	1.928	6.02
$[Cr_2$(D*p*-ClPhF)$_4]$ [31]	1.907	3.17
$[Cr_2$(D*m*,*m*-Cl_2PhF)$_4]$ [31]	1.916	4.55
$[Cr_2$(D*m*-(CF_3)PhF)$_4]$ [31]	1.902	2.52
$[Cr_2$(D*m*-AniF)$_4]$ [31]	1.918	1.06
$[Cr_2$(D*o*-TolF)$_4]$ [32]	1.925	3.56
$[Cr_2$(D*p*-(pyr)PhF)$_4]$ [33]	1.904	8.95
$[Cr_2$(D*p*-AniF)$_4]$ [34]	1.924	10.15

Table S11. Average Mo-Mo distances and $\chi_{Neq.\text{-}Mo\text{-}Mo\text{-}Neq.}$ dihedral angles for $[Mo_2(DArF)_4]$.

	**dMo-Mo / Å**	$\chi_{Neq.\text{-}Mo\text{-}Mo\text{-}Neq.}$ **/ deg**
$[Mo_2$(TPG)$_4]$ [35]	2.092	6.90
$[Mo_2$(D*p*-TolF)$_4]$ [36]	2.085	3.29
$[Mo_2$(DPhF)$_4]$ [37]	2.096	1.40
$[Mo_2$(D*m*,*m*-Cl_2PhF)$_4]$ [37]	2.097	0.50
$[Mo_2$(D*m*-ClPhF)$_4]$ [37]	2.096	0.42
$[Mo_2$(D*p*-AniF)$_4]$ [37]	2.096	1.04
$[Mo_2$(gua.)$_4]$ [38]	2.084	4.52
$[Mo_2$(D*p*-ClPhF)$_4]$ [39]	2.090	2.79
$[Mo_2$(D*p*-BrPhF)$_4]$ [40]	2.087	2.43
$[Mo_2$(D*o*-AniF)$_2$(2-pyridyl)$_2]$ [41]	2.107	0.58
$[Mo_2$(D*m*-AniF)$_4]$ [42]	2.102	0.38
$[Mo_2$(D*p*-(CCPh)-PhF)$_4]$ [43]	2.096	7.90
$[Mo_2$(D*p*,*m*,*o*-F_3PhF)$_4]$ [44]	2.102	3.96
$[Mo_2$(D*p*-FPhF)$_4]$ [44]	2.093	1.92
$[Mo_2$(D*m*,*m*-F_2PhF)$_4]$ [44]	2.099	0.59

[Mo_2(DF_5PhF)$_4$] [44]	2.100	4.45
[Mo_2(D*p*,*m*,*m*-F_3PhF)$_4$] [44]	2.092	0.35
[Mo_2(D*p*,*o*,*o*-F_3PhF)$_4$] [44]	2.102	4.40
[Mo_2(D*p*-(CF_3)PhF)$_4$] [44]	2.103	2.83
[Mo_2(D*m*,*m*-(CF_3)$_2$PhF)$_4$] [44]	2.096	0.30
[Mo_2(D*p*,*m*,*o*-F_3PhF)$_4$] [44]	2.099	3.00

Table S12. Average W-W distances and $\chi_{Neq.-W-W-Neq.}$ dihedral angles for [W_2(DArF)$_4$].

	**dW-W / Å**	**$\chi_{Neq.-W-W-Neq.}$ / deg**
[W_2(D*p*-TolF)$_4$] [29]	2.187	2.81
[W_2(D*m*,*m*-Cl_2PhF)$_4$] [45]	2.193	0.21
[W_2(D*m*,*m*-Cl_2PhF)$_4$] [46]	2.190	0.42
[W_2(D*p*-AniF)$_4$] [46]	2.196	0.25
[W_2(D*p*-ClPhF)$_4$] [47]	2.192	2.77
[W_2(D*p*-AniF)$_4$] [47]	2.196	1.08

Table S13. Average Rh-Rh distances and $\chi_{Neq.-Rh-Rh-Neq.}$ dihedral angles for [Rh_2(DArF)$_4$].

	**dRh-Rh / Å**	**$\chi_{Neq.-Rh-Rh-Neq.}$ / deg**
[Rh_2(D*p*-TolF)$_4$] [48]	2.434	16.75
[Rh_2(D*p*-TolF)$_4$] [49]	2.458	3.46
[Rh_2(DPB)$_4$] [50]	2.389	17.24
[Rh_2(D*m*,*m*-Cl_2PhF)$_4$] [51]	2.458	7.53
[Rh_2(D*m*-AniF)$_4$] [51]	2.452	17.44
[Rh_2(TPG)$_4$] [52]	2.408	17.42
[Rh_2(DPh-isonicotinamidate)$_4$] [53]	2.405	12.64
[Rh_2(DPh-*p*-(NH_2)benzamidinate)$_4$] [54]	2.395	17.60
[Rh_2(DPh-*p*-(NPh_2)benzamidinate)$_4$] [54]	2.392	16.51
[Rh_2(DPh-*p*-Br-benzamidinate)$_4$] [55]	2.401	12.41
[Rh_2(DPh-*p*-Br-benzamidinate)$_3$(DPh-(*p*-pyridin-*p*-yl)benzamidinate)] [55]	2.402	13.10
[Rh_2(DPh-*p*-Br-benzamidinate)$_2$(DPh-(*p*-pyridin-*p*-yl)benzamidinate)$_2$] [55]	2.398	15.11
[Rh_2(DPh-(*p*-pyridin-*p*-yl)benzamidinate)$_4$] [55]	2.404	11.75
[Rh_2(D*p*-TolF)$_4$] [56]	2.451	4.13

S.9. M-M BOND DISTANCES AND TORSION ANGLES IN [M_2Cl(ap)$_4$] COMPOUNDS (M = Ru, Rh)

Table S14. Average Ru-Ru bond distances and $\chi_{Neq.-Ru-Ru-Neq.}$ angles for [Ru_2Cl(ap)$_4$]-type compounds (ap = anilinopyridinate).

	**dRu-Ru / Å**	**$\chi_{Neq.-Ru-Ru-Neq.}$ / deg**
[Ru_2Cl(ap)$_4$] [57]	2.276	22.71
[Ru_2Cl(*o*-F-ap)$_4$] [58]	2.286	17.64
[Rh_2Cl(*o*,*m*-F_2ap)$_4$] [59]	2.283	18.59

$[Rh_2Cl(\mathit{o,o,p}\text{-}F_3ap)_4]$ [59]	2.296	24.01
$[Ru_2Cl(\mathit{o}\text{-}(CH_3)ap)_4]$ [59]	2.279	21.57
$[Rh_2Cl(\mathit{o,o}\text{-}F_2ap)_4]$ [59]	2.286	19.65
$[Ru_2Cl(\mathit{m}\text{-}(OCH_3)ap)_4]$ [60]	2.282	21.54
$[Ru_2Cl(a\text{-}\mathit{p}\text{-}(CH_3)p)_4]$ [61]	2.291	13.91
$[Rh_2Cl(\mathit{m,m}\text{-}bis(OCH_3)_2ap)_4]$ [62]	2.280	23.11
$[Ru_2Cl(a\text{-}\mathit{p}\text{-}(NH_2)p)_4]$ [63]	2.290	18.52

Table S15. Average Rh-Rh bond distances and $\chi_{Neq.\text{-}Rh\text{-}Rh\text{-}Neq.}$ angles for $[Rh_2Cl(ap)_4]$-type compounds (ap = anilinopyridinate).

	**dRh-Rh / Å**	$\chi_{Neq.\text{-}Rh\text{-}Rh\text{-}Neq.}$ **/ deg**
$[Rh_2Cl(ap)_4]$ [64]	2.406	23.39
$[Rh_2Cl(\mathit{o}\text{-}Fap)_4]$ [65]	2.413	21.38
$[Rh_2Cl(\mathit{o,o}\text{-}F_2ap)_4]$ [65]	2.416	24.68
$[Rh_2Cl(\mathit{o,o}\text{-}F_2ap)_4]$ [65]	2.420	20.80
$[Rh_2Cl(F_5\text{-}ap)_4]$ [65]	2.415	21.76

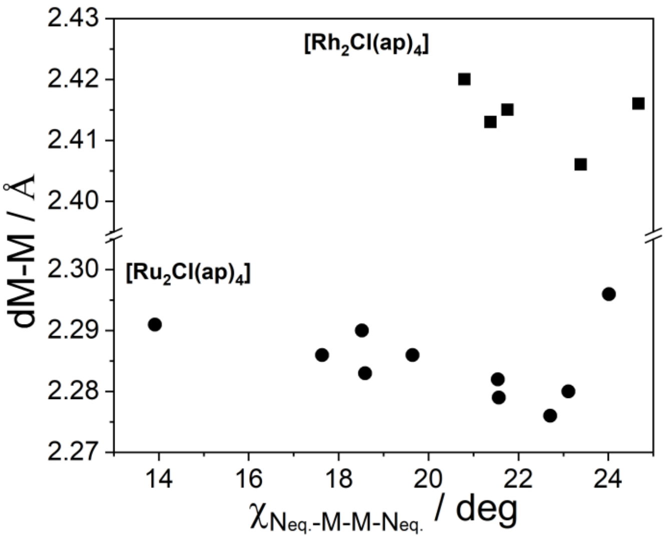

Figure S26. Variation of Rh-Rh (squares) and Ru-Ru (circles) bond distances with the $N_{eq.}$-M-M-$N_{eq.}$ dihedral angle in compounds with general formula $[M_2Cl(ap)_4]$ (ap = anilinopyridinate). The light dashed lines are guides to the eye. The distances and torsion values were obtained from the literature.

S.10. REFERENCES

[1] R. W. Mitchell, A. Spencer, G. Wilkinson, *J. Chem. Soc., Dalton Trans.* **1973**, (8), 846.

[2] R. M. Roberts, *J. Org. Chem.* **1949**, *14* (2), 277.

[3] F. A. Urbanos, **2002**.

[4] K. Momma, F. Izumi, *J. Appl. Crystallogr.* **2011**, *44* (6), 1272.

[5] P. Angaridis, F. A. Cotton, C. A. Murillo, D. Villagrán, X. Wang, *Inorg. Chem.* **2004**, *43* (26), 8290.

[6] S. Herrero, R. Jiménez-Aparicio, J. Perles, J. L. Priego, S. Saguar, F. A. Urbanos, *Green Chem.* **2011**, *13* (7), 1885.

[7] A. Inchausti, A. Terán, A. Manchado-Parra, A. de Marcos-Galán, J. Perles, M. Cortijo, R. González-Prieto, S. Herrero, R. Jiménez-Aparicio, *Dalton Trans.* **2022**, *51* (25), 9708.
[8] J. L. Bear, B. Han, S. Huang, K. M. Kadish, *Inorg. Chem.* **1996**, *35* (10), 3012.
[9] F. A. Cotton, T. Ren, *Inorg. Chem.* **1995**, *34* (12), 3190.
[10] P. Angaridis, F. A. Cotton, C. A. Murillo, D. Villagrán, X. Wang, *J. Am. Chem. Soc.* **2005**, *127* (14), 5008.
[11] W.-Z. Chen, F. A. Cotton, N. S. Dalal, C. A. Murillo, C. M. Ramsey, T. Ren, X. Wang, *J. Am. Chem. Soc.* **2005**, *127* (36), 12691.
[12] J. S. Pap, J. L. Snyder, P. M. Piccoli, J. F. Berry, *Inorg. Chem.* **2009**, *48* (20), 9846.
[13] G.-L. Xu, T. Ren, *Inorg.Chem.* **2006**, *45* (26), 10449.
[14] S. Leach, H. Scheraga, *J. Am. Chem. Soc.* **1960**, *82* (18), 4790.
[15] A. J. Basalla, B. S. Kendrick, *J. Pharm. Sci.* **2023**.
[16] A. R. Corcos, M. D. Roy, M. M. Killian, S. Dillon, T. C. Brunold, J. F. Berry, *Inorg. Chem.* **2017**, *56* (23), 14662.
[17] G. t. Te Velde, F. M. Bickelhaupt, E. J. Baerends, C. Fonseca Guerra, S. J. van Gisbergen, J. G. Snijders, T. Ziegler, *J. Comput. Chem.* **2001**, *22* (9), 931.
[18] M. Swart, F. M. Bickelhaupt, *J. Comput. Chem.* **2008**, *29* (5), 724.
[19] E. Van Lenthe, E. J. Baerends, *J. Comput. Chem.* **2003**, *24* (9), 1142.
[20] D. P. Chong, E. Van Lenthe, S. Van Gisbergen, E. J. Baerends, *J. Comput. Chem.* **2004**, *25* (8), 1030.
[21] M. Swart, *Chem. Phys. Lett.* **2013**, *580*, 166.
[22] A. Klamt, G. Schüürmann, *J. Chem. Soc., Perkin Trans. 2* **1993**, (5), 799.
[23] B. B. Averkiev, D. G. Truhlar, *Catal. Sci. Technol.* **2011**, *1* (8), 1526.
[24] J. E. Klein, B. Dereli, L. Que, C. J. Cramer, *Chem. Comm.* **2016**, *52* (69), 10509.
[25] E. v. Lenthe, E.-J. Baerends, J. G. Snijders, *J. Chem. Pys.* **1993**, *99* (6), 4597.
[26] M. Axton, A. Baak, N. Blomberg, J.-W. Boiten, L. B. da Silva Santos, P. E. Bourne, J. Bouwman, A. J. Brookes, T. Clark, *Sci. Data* **2016**, *3*, 160018.
[27] F. A. Cotton, L. M. Daniels, C. A. Murillo, *Angew. Chem., Int. Ed. Engl.* **1992**, *31* (6), 737.
[28] F. A. Cotton, E. A. Hillard, C. A. Murillo, X. Wang, *Inorg. Chem.* **2003**, *42* (19), 6063.
[29] F. A. Cotton, T. Ren, *J. Am. Chem. Soc.* **1992**, *114* (6), 2237.
[30] F. A. Cotton, C. A. Murillo, I. Pascual, *Inorg. Chem.* **1999**, *38* (9), 2182.
[31] K. M. Carlson-Day, J. L. Eglin, C. Lin, L. T. Smith, R. J. Staples, D. O. Wipf, *Polyhedron* **1999**, *18* (6), 817.
[32] F. A. Cotton, L. M. Daniels, C. A. Murillo, P. Schooler, *J. Chem. Soc., Dalton Trans.* **2000**, (13), 2007.
[33] F. A. Cotton, L. M. Daniels, P. Huang, C. A. Murillo, *Inorg. Chem.* **2002**, *41* (2), 317.
[34] F. A. Cotton, Z. Li, C. A. Murillo, Precursors for Assembly of Supramolecules Containing Quadruply Bonded Cr_{24+} Units: Systematic Preparation of Cr_2(formamidinate)$_n$(acetate)$_{4-n}$ (n= 2–4). *Chem. - Eur. J.* **2007**, 3509-3513.
[35] F. Cotton, T. Inglis, M. Kilner, T. Webb, *Inorg. Chem.* **1975**, *14* (9), 2023.
[36] F. A. Cotton, X. Feng, M. Matusz, *Inorg. Chem.* **1989**, *28* (3), 594.
[37] C. Lin, J. D. Protasiewicz, E. T. Smith, T. Ren, *Inorg. Chem.* **1996**, *35* (22), 6422.
[38] P. J. Bailey, S. F. Bone, L. A. Mitchell, S. Parsons, K. J. Taylor, L. J. Yellowlees, *Inorg. Chem.* **1997**, *36* (5), 867.

[39] M. A. Lynn, H. D. Selby, M. D. Carducci, M. A. Bruck, C. Grittini, D. L. Lichtenberger, *Acta Cryst. E* **2001**, *57* (2), m57-m58.
[40] M. A. Lynn, H. D. Selby, M. D. Carducci, M. A. Bruck, C. Grittini, D. L. Lichtenberger, *Acta Cryst. E* **2001**, *57* (2), m70-m71.
[41] Y.-Y. Wu, Y.-C. Kao, J.-D. Chen, C.-H. Hung, *Inorg. Chim. Acta* **2004**, *357* (4), 1002.
[42] Y.-Y. Wu, C.-W. Yeh, Z.-K. Chan, C.-H. Lin, C.-H. Yang, J.-D. Chen, J.-C. Wang, *J. Mol. Struct.* **2008**, *890* (1-3), 48.
[43] C. Jiang, P. J. Young, C. B. Durr, T. F. Spilker, M. H. Chisholm, *Inorg. Chem.* **2016**, *55* (12), 5836.
[44] I. A. Squire, C. A. Goult, B. C. Thompson, E. Alexopoulos, A. C. Whitwood, T. F. Tanner, L. A. Wilkinson, *Inorg. Chem.* **2022**, *61* (48), 19144.
[45] K. M. Carlson-Day, J. L. Eglin, L. T. Smith, R. J. Staples, *Inorg. Chem.* **1999**, *38* (9), 2216.
[46] J. L. Eglin, L. T. Smith, R. J. Staples, *Inorg. Chim. Acta* **2003**, *351*, 217.
[47] F. A. Cotton, J. P. Donahue, M. B. Hall, C. A. Murillo, D. Villagrán, *Inorg. Chem.* **2004**, *43* (22), 6954.
[48] P. Piraino, G. Bruno, S. Schiavo, F. Laschi, P. Zanello, *Inorg. Chem.* **1987**, *26* (14).
[49] J. L. Bear, C. Yao, R. Lifsey, J. Korp, K. Kadish, *Inorg. Chem.* **1991**, *30* (2), 336.
[50] L. He, C. Yao, M. Naris, J. Lee, J. Korp, J. Bear, *Inorg. Chem.* **1992**, *31* (4), 620.
[51] T. Ren, C. Lin, E. J. Valente, J. D. Zubkowski, *Inorg. Chim. Acta* **2000**, *297* (1-2), 283.
[52] D. A. Lutterman, N. N. Degtyareva, D. H. Johnston, J. C. Gallucci, J. L. Eglin, C. Turro, *Inorg. Chem.* **2005**, *44* (15), 5388.
[53] D. Chartrand, G. S. Hanan, *Chem. Comm.* **2008**, (6), 727.
[54] M. W. Cooke, M.-P. Santoni, G. S. Hanan, A. Proust, B. Hasenknopf, *Dalton Trans.* **2009**, (19), 3671.
[55] D. Chartrand, G. S. Hanan, *J. Phys. Chem. A* **2014**, *118* (45), 10340.
[56] S. De Doncker, A. Casimiro, I. A. Kotze, S. Ngubane, G. S. Smith, *Inorg. Chem.* **2020**, *59* (17), 12928.
[57] A. R. Chakravarty, F. A. Cotton, D. A. Tocher, *Inorg. Chem.* **1985**, *24* (2), 172.
[58] J. L. Bear, J. Wellhoff, G. Royal, E. V. Caemelbecke, S. Eapen, K. M. Kadish, *Inorg. Chem.* **2001**, *40* (10), 2282.
[59] K. M. Kadish, L.-L. Wang, A. Thuriere, E. Van Caemelbecke, J. L. Bear, *Inorg. Chem.* **2003**, *42* (3), 834.
[60] G.-L. Xu, A. Cordova, T. Ren, *J. Clust. Sci.* **2004**, *15*, 413.
[61] K. M. Kadish, M. Nguyen, E. Van Caemelbecke, J. L. Bear, *Inorg. Chem.* **2006**, *45* (15), 5996.
[62] B. Xi, G.-L. Xu, J.-W. Ying, H.-L. Han, A. Cordova, T. Ren, *Jo. Organomet. Chem.* **2008**, *693* (8-9), 1656.
[63] N. V. Naidu, H. Arman, Y. Deng, X. Wei, *J. Coord. Chem.* **2014**, *67* (18), 3006.
[64] J. Bear, C. Yao, L. Liu, F. Capdevielle, J. Korp, T. Albright, S. Kang, K. Kadish, *Inorg. Chem.* **1989**, *28* (7), 1254.
[65] K. M. Kadish, T. D. Phan, L. Giribabu, E. Van Caemelbecke, J. L. Bear, *Inorg. Chem.* **2003**, *42* (26), 8663.
